# Factors linked to successful brain magnetic resonance imaging scans and data quality in autistic individuals across the functioning spectrum

**DOI:** 10.1192/bjo.2026.12035

**Published:** 2026-07-14

**Authors:** Lin-Wan Huang, Yi Ran Zhou, Chun-Hung Yeh, Hsing-Chang Ni, Benoit H. Mulsant, Muhammad Ishrat Husain, Jung-Chi Chang, En-Nien Tu, Mei-Yun Hsu, Yu-Yu Wu, Tai-Li Chou, Susan Shur-Fen Gau, Hsiang-Yuan Lin

**Affiliations:** Mood and Anxiety Ambulatory Services, Centre for Addiction and Mental Health, Toronto, Canada; Department of Psychiatry, University of Toronto, Toronto, Canada; Azrieli Adult Neurodevelopmental Centre, Centre for Addiction and Mental Health, Toronto, Canada; Department of Medical Imaging and Radiological Sciences, Chang Gung University, Taoyuan, Taiwan; Department of Psychiatry, Chang Gung Memorial Hospital Linkou, Taoyuan, Taiwan; College of Medicine, Chang Gung University, Taoyuan, Taiwan; Campbell Family Mental Health Research Institute, Centre for Addiction and Mental Health, Toronto, Canada; Department of Psychiatry, National Taiwan University Hospital and College of Medicine, Taipei, Taiwan; Department of Psychiatry, Keelung Chang Gung Memorial Hospital, Keelung, Taiwan; YuNing Clinic, Taipei, Taiwan; Department of Psychology, National Taiwan University, Taipei, Taiwan

**Keywords:** Autism spectrum disorder, intellectual disability, magnetic resonance imaging (MRI), minimally verbal, data quality

## Abstract

**Background:**

Neuroimaging research in autism often excludes individuals with co-occurring intellectual impairment or minimally verbal status, limiting the generalisability of findings. Understanding magnetic resonance imaging (MRI) scan success predictors in a representative autistic sample is crucial for equitable research.

**Aims:**

This study identified factors predicting successful brain MRI acquisition and data quality in diverse autistic individuals, focusing on including those with intellectual impairment or minimally verbal status.

**Method:**

A total of 122 participants (83 autistic individuals (27 with intellectual impairment, 19 with minimally verbal status) and 39 typically developing controls) received multi-modal brain MRI scans (including structural, resting-state functional and diffusion MRI). Scan success, assessed using both binary criteria and quantitative data-quality metrics, was related to participant characteristics.

**Results:**

Although overall scan success was high, specific factors differentiated success within subgroups. Key factors contributing to scan success included age, non-verbal intelligence and attention-deficit hyperactivity disorder (ADHD) symptoms. Older participants, those with fewer ADHD symptoms and those with higher non-verbal intelligence were more likely to achieve successful scans, regardless of autism diagnosis. Higher data quality, particularly in structural and functional MRI, was associated with higher intelligence, better adaptive functioning, fewer autistic and ADHD symptoms, and fewer behavioural problems.

**Conclusions:**

Identifying these factors is key to designing more inclusive and effective neuroimaging protocols. This work paves the way for more comprehensive research into the neurobiology of the full autism spectrum, and offers insights for improving the clinical MRI experience for autistic individuals with diverse support needs. Individualised strategies may also be useful in clinical settings, helping to improve the experience of MRI scanning for autistic individuals.

Understanding the neurobiological underpinnings of autism spectrum condition (ASC)^a^ ^,^^1^ through neuroimaging is a major research focus.^2–4^ However, a significant limitation in the field is the frequent exclusion of autistic individuals who have co-occurring intellectual disability or are minimally verbal. This practice not only skews our understanding toward a segment of the autistic population with fewer support needs, but also hampers the development of tailored supports for those who may require them most. This exclusion results in a fragmented understanding of ASC, potentially leading to clinical insights and interventions that are not applicable or effective for a substantial portion of autistic individuals who require significant support. Indeed, it is estimated that 25–30% of children with ASC are minimally verbal,^5–8^ and a significant proportion (e.g. 58.3% in a USA sample^8^) have co-occurring intellectual impairment (II), an umbrella term we use in this paper to include ID and borderline intellectual functioning. Despite their prevalence, these individuals have historically been underrepresented in neuroimaging research,^5,9^ leading to biased samples and limited generalisability of findings.

Autism itself is a neurodevelopmental condition characterised by differences in social communication and the presence of restricted, repetitive patterns of behaviour, interests or activities,^10^ affecting approximately 1% of the population with a 4–5:1 male:female ratio.^11^ This inherent diversity is reflected in varied brain structure, function and connectivity, particularly in networks crucial for socio-emotional cognition, sensorimotor integration and executive function.^2^ Although brain magnetic resonance imaging (MRI) is a powerful tool for investigating these neural correlates, inconsistencies in findings persist, partly because of limited sample sizes and methodological variability.^2,12^ These challenges are amplified when attempting to include autistic individuals with intellectual impairment or minimally verbal status. Acquiring high-quality MRI data from these participants can be impeded by difficulties with communication, understanding instructions and heightened sensory sensitivities to the scanner environment (e.g. noise, confinement).^13,14^

Nevertheless, several studies have demonstrated the feasibility of acquiring MRI data in these populations with careful preparation and adapted protocols. For instance, Nordahl et al^15^ successfully scanned 17 autistic participants with intellectual impairment using mock scanner familiarisation. Similarly, Smith et al^16^ reported successful completion of an hour-long positron emission tomography/MRI protocol in 18 out of 19 autistic participants across the intellectual spectrum. These pioneering efforts underscore the potential for more inclusive neuroimaging research.

While strategies to improve MRI scan success in autistic individuals have been explored,^13^ the specific cognitive, behavioural and demographic factors that contribute to successful data acquisition, particularly across the full spectrum of autism and using nuanced measures of data quality, remain largely unknown. Much previous research has relied on binary ‘pass/fail’ criteria for scan success, which may not fully capture data-quality variations crucial for robust analysis.^17^

Therefore, the current study sought to investigate the association of commonly collected demographic, cognitive and behavioural measures with both binary and quantitative metrics of scan success and data quality in a multi-modal brain MRI protocol in a diverse sample of autistic individuals, including those with co-occurring intellectual impairment and/or minimally verbal status. Based on previous literature,^17,18^ we hypothesised that older age, higher intelligence and lower severity of co-occurring attention-deficit hyperactivity disorder (ADHD) symptoms would be positive predictors of scan success and data quality across the autism spectrum, while also exploring a range of other participant characteristics.

## Method

### Participants

Data used in this paper were obtained as part of a study approved by the Research Ethics Committee of the National Taiwan University Hospital, Taipei, Taiwan (#201512238RINC), which looked at the behaviour, cognition and brain organisation of autistic individuals with intellectual disability or minimally verbal status. Written informed consent was obtained from participants and their parents. For participants incapable of consenting, assent was obtained, and a substitute decision maker completed the informed consent on their behalf. A total of 125 participants were initially recruited for the study. Three participants did not attempt the MRI scanning portion of the protocol because of personal reasons, and were excluded from the analysis. Of the remaining 122 participants, 83 were autistic individuals referred from psychiatric out-patient clinics at the National Taiwan University Hospital, the Yuning Psychiatric Clinic and the Linkou Chang Gung Memorial Hospital between December 2016 and August 2019. A total of 39 age-matched typically developing controls (TDCs) were recruited from neighbourhoods of similar socioeconomic status, during the same time frame. Participants were between 7 and 30 years old at the time of MRI scans.

### Clinical assessments and eligibility

ASC diagnoses were first clinically made by any of the child psychiatrists (H.-C.N., Y.-Y.W., S.S.-F.G. or H.-Y.L.) based on the DSM-5, and further confirmed on enrolment by the senior author (H.-Y.L.), using the Autism Diagnostic Observation Schedule – Second Edition (ADOS-2)^19^ and the Autism Diagnostic Interview – Revised (ADI-R).^20^ For the ADOS-2, an appropriate module was chosen for each participant based on age and functional level, and a standardised calibrated severity score (CSS) was calculated.^21,22^ For the ADI-R, all autistic participants were diagnostically confirmed with the diagnostic algorithm, whereas the total scores based on the current algorithm were presented and used for the data analysis. TDCs were clinically assessed to ensure that they did not have any psychiatric diagnoses. Parents of all participants, including TDCs, were interviewed by H.-Y.L. for co-occurring psychiatric conditions with the Kiddie-Schedule for Affective Disorders and Schizophrenia-Epidemiological Version.^23^ Participants were excluded from the ASC group only if they had an acute or unstable medical illness; a history of psychosurgery or head trauma; active grand mal seizures in the past year; known genetic causes contributing to ASC, intellectual impairment or minimally verbal status (determined via comprehensive medical history and caregiver reports of prior clinical genetic testing; systematic genomic sequencing was not performed for this study); lifetime history of bipolar, psychotic or substance use disorders; current suicidal ideation or pregnancy. Participants with co-occurring ADHD or adjustment disorder were included in our analysis unless they met other exclusion criteria. Finally, participants taking stimulants for ADHD were asked to withhold medication starting 24 h before their MRI scan. Supplementary Table 1 presents details of medication uses and co-occurring psychiatric conditions in autistic individuals. All participants underwent standard MRI safety screening before the scan, including checks for metallic implants, pacemakers and claustrophobia.

### Cognitive and behavioural assessments

Four measures were used to establish a functioning profile for all participants. Cognitive function was assessed with the Wechsler Intelligence Scale for Children – Fourth Edition (WISC-IV)^24^ or the Wechsler Adult Intelligence Scale – Fourth Edition (WAIS-IV)^25^ (at the age cut-off of 16 years), and the Leiter International Performance Scale – Revised (Leiter-R).^26^ Adaptive function was quantified with the Adaptive Behavior Composite of the Vineland Adaptive Behavior Scales (VABS ABC).^27^ Executive function was measured with the Global Executive Composite of the Behavior Rating Inventory of Executive Function (BRIEF-GEC).^28^ Higher scores on the WISC-IV, WAIS-IV, Leiter-R and VABS ABC indicate better function, whereas higher scores on the BRIEF-GEC reflect worse executive function.

Five measures were used to establish a behaviour profile for all participants. Autistic symptoms were estimated with the Social Responsiveness Scale (SRS) for reciprocal social behaviour,^29^ the Repetitive Behavior Scale – Revised (RBS-R) for repetitive behaviour severity,^30^ and the Short Sensory Profile (SSP) for sensory processing difficulties.^31^ The Aberrant Behavior Checklist (ABC) was used for estimating behaviour and emotion problems.^32^ Because existing literature suggests that around 40–50% of autistic individuals have co-occurring ADHD,^33^ the Swanson, Nolan, and Pelham-IV Questionnaire (SNAP-IV)^34^ was administered to assess ADHD symptoms. Higher scores on all these five behavioural measures reflect more severe difficulties. Measures for adaptive and executive function, autistic symptoms and other behaviour issues were rated by participants’ parents or caregivers.

### Grouping

**Fig. 1:**
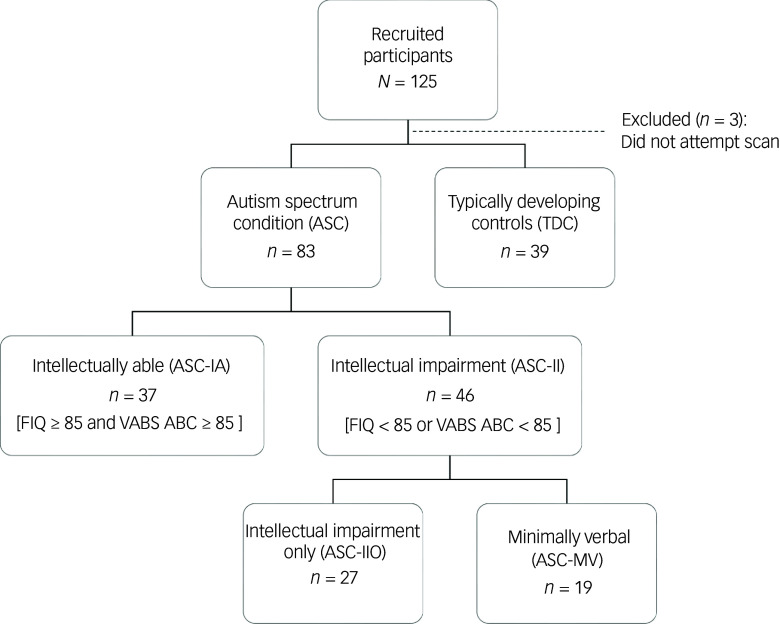
Fig. 1 long description. Participant recruitment and data inclusion flowchart. FIQ, Wechsler Full-Scale Intelligence Quotient; VABS ABC, Adaptive Behavior Composite of the Vineland Adaptive Behavior Scales.

To better explore the heterogeneity of ASC, we adopted three grouping strategies: ASC versus TDCs (grouping 1; Supplementary Table 2), intellectually able ASC (ASC-IA) versus ASC with intellectual impairment (ASC-II) versus TDCs (grouping 2; Table 1), and ASC-IA versus ASC-II only (ASC-IIO) versus ASC with II and minimally verbal status (ASC-MV) versus TDCs (grouping 3; Supplementary Table 3). Specifically, first, we divided autistic participants into two subgroups by using the Wechsler Full-Scale Intelligence Quotient (FIQ) and the VABS ABC. The first subgroup is ASC-IA (*n* = 37), characterised by an FIQ of ≥85 and VABS ABC score of ≥85. The second subgroup is ASC-II (*n* = 46), characterised by an FIQ score <85 or VABS ABC score <85. The FIQ cut-off was set at 85 because autistic children with borderline intellectual functioning (between 70 and 84) exhibit similar developmental trajectories to autistic children with intellectual disability.^35^ A stringent VABS ABC cut-off was included in the definition because FIQ alone is a poor predictor of functional ability and long-term outcomes, especially in ASC-IA.^36^ ASC-II was then further divided into two groups: ASC-IIO (*n* = 27) and ASC-MV (*n* = 19). Participants with ASC-MV spoke fewer than 30 words or phrases as reported by their parents and judged by a child psychiatrist (H.-Y.L. or Y.-Y.W.).^5^ The participant recruitment and hierarchical subgrouping process is illustrated in Fig. 1.

**Table 1:** Demographic and clinical profiles of participants (grouping 2) Table 1 long description.

Variable	ASC-II (*n* = 46)	ASC-IA (*n* = 37)	TDCs (*n* = 39)	Statistics^a^	*Post hoc* ^b^
Demographics					
Age, years	15 [9]	15 [9]	17 [8]	*H* = 1.24, *p* = 0.538	—
Age range, years	7–30	7–26	8–30		
Gender (male/female)	42/4	32/5	31/8	*X* ^*2*^ = 2.47, *p* = 0.291	—
Medication (yes/no)	23/23	21/16	0/39	*X* ^*2*^ = 32.74, *p* < 0.001	ASC-II, ASC-IA > TDC
Comorbidity (yes/no)	31/15	24/13	0/39	*X* ^*2*^ = 47.11, *p* < 0.001	ASC-II, ASC-IA > TDC
ASC profile					
ADOS-2 CSS^c^	6 [3]	5 [4]	—	*H = 7.53, p* = 0.003	ASC-II > ASC-IA
ADI-R total	25 [7]	21 [8]	—	*H* = 11.44, *p* < 0.001	ASC-II > ASC-IA
SRS total	97 [32]	85 [36]	19 [12]	*H* = 76.90, *p* < 0.001	ASC-II, ASC-IA > TDC
SSP total	34 [30]	30 [35.5]	3 [8]	*H* = 59.08, *p* < 0.001	ASC-II, ASC-IA > TDC
RBS-R total	24 [24]	24.5 [24.5]	1 [5]	*H* = 61.22, *p* < 0.001	ASC-II, ASC-IA > TDC
Functional profile					
FIQ^d^	67 [26]	102 [18]	113 [15]	*H* = 77.00, *p* < 0.001	ASC-II < ASC-IA, TDC
Leiter-R NVFIQ	73 [45]	119 [23]	121 [12]	*H* = 65.33, *p* < 0.001	ASC-II < ASC-IA, TDC
VABS ABC	64 [13]	90 [21]	111.5 [25.8]	*H* = 85.27, *p* < 0.001	ASC-II < ASC-IA < TDC
BRIEF-GEC	154 [43]	155 [30]	87 [27]	*H* = 66.01, *p* < 0.001	ASC-II, ASC-IA > TDC
Behavioural profile					
ABC total	52 [60]	34 [43]	0 [5]	*H* = 60.58, *p* < 0.001	ASC-II, ASC-IA > TDC
SNAP-IV total	23 [17]	22 [18]	3 [9]	*H* = 55.47, *p* < 0.001	ASC-II, ASC-IA > TDC
MRI quality metrics					
sMRI – IQR	88.30 [1.42]	88.34 [1.59]	88.98 [0.50]	*H* = 18.09, *p* < 0.001	ASC-II, ASC-IA < TDC
fMRI – mean framewise displacement (mm)	0.50 [1.18]	0.50 [0.55]	0.26 [0.30]	*H* = 12.88, *p* = 0.002	ASC-II, ASC-IA > TDC
fMRI – accepted/rejected component ratio	0.13 [0.19]	0.15 [0.18]	0.22 [0.18]	*H* = 1.15, *p* = 0.563	—
dMRI – mean relative root-mean-square (mm)	0.45 [0.38]	0.35 [0.22]	0.32 [0.17]	*H* = 8.61, *p* = 0.013	ASC-II > TDC

All continuous variables are presented as median [interquartile range]. ASC-II, autism spectrum condition with intellectual impairment only or autism spectrum condition with intellectual impairment and minimally verbal status; ASC-IA, intellectually-able autism spectrum condition; TDCs, typically developing controls; ASC, autism spectrum condition; ADOS-2 CSS, Autism Diagnostic Observation Schedule-Second Edition, calibrated severity score; ADI-R, Autism Diagnostic Interview-Revised, total score based on the current algorithm; SRS, Social Responsiveness Scale; SSP, Short Sensory Profile; RBS-R, Repetitive Behavior Scale-Revised; FIQ, Wechsler Intelligence Scale, Full-scale Intelligence Quotient; Leiter-R NVFIQ, Leiter International Performance Scale-Revised, Non-verbal Full Intelligence Quotient; VABS ABC, Vineland Adaptive Behavior Scale, Adaptive Behavior Composite; BRIEF-GEC, Behavior Rating Inventory of Executive Function, Global Executive Composite; ABC, Aberrant Behavior Checklist; SNAP-IV, Swanson, Nolan, and Pelham-IV Questionnaire; IQR, CAT12’s image quality rating.

a. Chi-squared test was used for gender, medication and comorbidity variables; Kruskal–Wallis test was used for all other variables.

b. Pairwise comparisons using the chi-squared test followed by Bonferroni correction were performed for gender, medication and comorbidity variables; the *post hoc* Dunn–Bonferroni test was used for all other variables.

c An appropriate ADOS-2 module was selected for each participant as determined by a child psychiatrist.

d. Wechsler Adult Intelligence Scale, Fourth Edition was used for participants aged 16 years and above; Wechsler Intelligence Scale for Children, Fourth Edition was used for participants younger than 16 years.

### MRI acquisition

MRI data were collected on a Siemens MAGNETOM Prisma 3-T MRI with a 64-channel phased-array head/neck coil, in the order of approximately 5 min T1-weighted structural MRI (sMRI), 7 min resting-state functional MRI (rsfMRI) and approximately 8 min diffusion MRI (dMRI). High-resolution MPRAGE T1-weighted sMRI were acquired using the following parameters: repetition time (TR) = 2000 ms, echo time (TE) = 2.43 ms, inversion time = 920 ms, flip angle (FA) = 9°, acquisition matrix = 256 × 256, slice thickness = 0.9 mm, in-plane resolution = 0.9 mm isotropic. To accelerate the acquisition, fMRI and dMRI were obtained using the simultaneous multi-slice (or multiband) echo-planar imaging (EPI) sequence developed at the Centre for Magnetic Resonance Research, University of Minnesota.^37^ To optimise fMRI data quality, we used a multi-echo EPI sequence with multiband acceleration, enabling faster acquisition, reducing signal drop-out and enhancing the blood oxygen level-dependent (BOLD) contrast-to-noise ratio. Multiband multi-echo fMRI acquisition adopted the following parameters: TR/TE = 995/14.2, 35.25, 56.3 ms (three echoes), field of view = 21 cm, matrix size = 80 × 80 with slice thickness = 2.6 mm (2.6 × 2.6 × 2.6 mm voxel size) with 0.156 mm spacing between slices, 48 slices with multiband factor = 4 (total slices 48), FA = 52°, and partial Fourier factor = 0.85, in-plane acceleration = 2. Multi-shell diffusion-weighted images were acquired using the following parameters: 2.2 mm isotropic voxel, TR/TE = 2238/86 ms, multiband acceleration factor = 4, number of diffusion gradient directions = 19/30/90/60 at *b* = 0/350/1000/3000 s/mm^2^, respectively.

### Support procedures during MRI scans

To enhance participant comfort and cooperation during MRI scans, we implemented a comprehensive support protocol adapted from a published study.^38^ This protocol included a preliminary evaluation by clinicians to assess the potential for MRI adherence, particularly for individuals with ASC and intellectual impairment. Before the scan, participants and their parents were provided with videos and recordings of scanner sounds to familiarise them with the MRI environment. An optional mock scanner session was also offered to further alleviate anxiety; this was utilised by 25 participants (13 for ASC-MV, eight for ASC-IIO, four for ASC-IA). On the day of the scan, participants were gradually acclimated to the scan room and equipment. Comfort measures, such as allowing personal clothing, physical contact with a companion and the use of comfort items, were employed throughout the scanning process. Additionally, the movie paradigm ‘Inscapes’^39^ was presented during sMRI and dMRI acquisitions to enhance adherence. Verbal communication with either the participant or their caregiver was maintained to ensure physical and psychological well-being throughout the procedure.

### MRI preprocessing and definition of successful scans

Preprocessing and quality control were conducted separately for each modality, combining (a) visual inspection for gross artifacts and (b) a quantitative summary metric used both to define scan success and index continuous data quality.

For sMRI, T1-weighted images were processed in the Computational Anatomy Toolbox (CAT12, version CAT12.9 (2550), Christian Gaser and Robert Dahnke, Structural Brain Mapping Group, Jena University Hospital, Jena, Germany; neuro-jena.github.io/cat/)^40^ running in Statistical Parametric Mapping 12 (SPM12; Wellcome Centre for Human Neuroimaging, University College London, UK; fil.ion.ucl.ac.uk/spm/) on MATLAB R2022a (MathWorks Inc, Natick, Massachusetts, USA; https://www.mathworks.com) to undertake voxel-based morphometry. Images first passed expert visual quality control (Human Connectome Project rating^41^ ‘fair’ or better) and were then required to exceed CAT12’s image quality rating (IQR) threshold (>70), a composite score derived from weighted measures of noise, inhomogeneity and resolution.^40^ A higher IQR score indicates better sMRI data quality.

For rsfMRI, multiband multi-echo fMRI images were denoised using tedana^42^ with multi-echo independent component analysis (ME-ICA),^43–46^ which separates BOLD-like (‘accepted’) from non-BOLD (‘rejected’) components by using echo-time dependence. We calculated the ratio of accepted to rejected independent components for each participant. However, as the total number of components is data-driven and varies by participant, we used this primarily as a metric of algorithmic stability and consistency of the denoising process across groups rather than a strict cut-off for scan success.^43–45^ rsfMRI scans were considered successful if they were free of major artifacts (e.g. severe signal drop-out or ghosting) in the raw fMRI data and the mean framewise displacement was <0.5 mm.^47^ We selected framewise displacement because it is widely reported, directly interpretable as head-motion magnitude,^48^ and provides a conservative motion summary even after ME-ICA denoising. The 0.5 mm threshold was selected *a priori* to balance inclusivity and data quality in a developmentally and clinically diverse sample.^49^

For dMRI, the diffusion data^50^ were preprocessed using MRtrix3 (version 3.0.2, Florey Institute of Neuroscience and Mental Health, Melbourne, Australia, and King’s College London, UK; mrtrix.org)^51^ and FSL (version 6.0.5, Analysis Group at the Oxford Centre for Functional MRI of the Brain (FMRIB), Oxford, UK; fsl.fmrib.ox.ac.uk/fsl/).^52^ dMRI scans were considered successful if raw diffusion-weighted images were free of major artifacts and mean intervolume motion (relative root-mean-square, displacement) was <1 mm.

Average framewise displacement and average relative root-mean-square are summarised estimates of head motion levels during rsfMRI and dMRI scans, respectively, with higher values corresponding to lower data quality. Visual quality control was performed by single expert raters to ensure internal consistency. sMRI and fMRI data were inspected by H.-Y.L. (>10 years of multi-modal MRI experience), and dMRI data were inspected by C.-H.Y. (>15 years of dMRI expertise).

### Statistics

As several measures/variables violated the normality assumption based on the Shapiro–Wilk test, we adopted non-parametric statistics throughout using SPSS 25.0 (SPSS Inc., Chicago, Illinois, USA). The Kruskal–Wallis *H*-test with *post hoc* Dunn–Bonferroni was used to make pairwise comparisons between participants groups for continuous demographic and clinical variables, whereas chi-squared tests with *post hoc* Bonferroni correction were used to identify significant differences in categorical variables (Table 1 and Supplementary Tables 2 and 3). The chi-squared test was used to highlight any differences in MRI success rates between participant groups, and significant hits were followed up with *post hoc* Fisher’s exact test and Bonferroni correction (Table 2). Categorical differences in clinical characteristics of participants completing all scans successfully versus unsuccessfully within each group were identified using the Mann–Whitney *U*-test (Table 3 and Supplementary Tables 4 and 5). Finally, the dimensional relationships between the image quality metrics (IQR for sMRI; average framewise displacement for rsfMRI; average relative root-mean-square for dMRI) and clinical, cognitive and behavioural variables, respectively, were established using the Spearman’s rank correlation (Table 4). The relationship between ADI-R total score or ADOS-2 CSS and image quality was computed within the ASC group only, whereas all other relationships were computed using pooled ASC and TDC data. Unless otherwise indicated, we applied the Benjamini–Hochberg method to control the false discovery rate ((FDR) the corrected alpha level expressed as *q*)^53^ for multiple tests in each set of analyses. Given the wide age range in this cohort, age was examined explicitly as a predictor of scan success and as a correlate of each modality’s quality metric. However, because diagnostic subgroups were statistically age-matched (Table 1 and Supplementary Tables 2 and 3) and the non-normal distribution of quality metrics necessitated non-parametric analyses, we did not fit additional multivariable models with age as a covariate. We acknowledge potential residual developmental confounding as a limitation.

**Table 2:** Comparison of MRI success rates by scan type between various subgroupings of participants with autism spectrum condition and typically developing controls Table 2 long description.

Grouping	Groups	Success criteria											
		sMRI			rsfMRI			dMRI			Completion of T1, rsfMRI and dMRI		
		Successful? (yes/no)	Statistics^a^	*Post hoc* ^b^	Successful? (yes/no)	Statistics^a^	*Post hoc* ^b^	Successful? (yes/no)	Statistics^a^	*Post hoc* ^b^	Successful? (yes/no)	Statistics^a^	*Post hoc* ^b^
Grouping 1	ASC	73/10	*χ* ^2^ = 5.12, *q* = 0.024	ASC < TDC	45/37^c^	*χ* ^2^ = 10.20, *q* = 0.003	ASC < TDC	67/14^c,d^	*χ* ^2^ = 5.22, *q* = 0.032	ASC < TDC	45/38	*χ* ^2^ = 10.20, *q* = 0.003	ASC < TDC
	TDC	39/0			33/6			38/1			33/6		
Grouping 2	ASC-II	37/9	*χ* ^2^ = 12.87, *q* = 0.003	ASC-II < ASC-IA < TDC	21/24^c^	*χ* ^2^ = 13.14, *q* = 0.004	ASC-II < ASC-IA < TDC	33/12^c^	*χ* ^2^ = 12.96, *q* = 0.001	ASC-II < ASC-IA, TDC	21/25	*χ* ^2^ = 13.14, *q* = 0.003	ASC-II < ASC-IA < TDC
	ASC-IA	36/1			24/13			34/2^d^			24/13		
	TDC	39/0			33/6			38/1			33/6		
Grouping 3	ASC-MV	12/7	*χ* ^2^ = 25.71, *q* < 0.001	ASC-MV < ASC-IIO < ASC-IA, TDC	4/14^c^	*χ* ^2^ = 20.96, *q* = 0.002	ASC-MV < ASC-IIO, ASC-IA, TDC	9/9^c^	*χ* ^2^ = 26.46, *q* = 0.001	ASC-MV < ASC-IIO, ASC-IA, TDC	4/15	*χ* ^2^ = 20.96, *q* = 0.002	ASC-MV < ASC-IIO, ASC-IA, TDC
	ASC-IIO	25/2			17/10			24/3			17/10		
	ASC-IA	36/1			24/13			34/2^d^			24/13		
	TDC	39/0			33/6			38/1			33/6		

MRI, magnetic resonance imaging; sMRI, structural MRI (T1-weighted image); rsfMRI, resting-state functional MRI; dMRI, diffusion MRI; ASC, autism spectrum condition (group includes both ASC-IA and ASC-II); TDC, typically developing controls; ASC-II, both ASC-MV and ASC-IIO; ASC-IA, intellectually able autism spectrum condition; ASC-MV, autism spectrum condition with intellectual impairment and minimally verbal status; ASC-IIO, autism spectrum condition with intellectual impairment only.

a. Chi-squared test was performed followed by Benjamini–Hochberg procedure to give false discovery rate-adjusted *p*-values (*q*-values), calculated for all comparison conditions (12 conditions).

b. Pairwise comparisons were completed using Fisher’s exact test followed by Bonferroni method.

c. One participant in the ASC-MV group was omitted because of scan parameter errors during rsfMRI and dMRI.

d. One participant in the ASC-IA group omitted because of scan parameter errors during dMRI.

**Table 3:** Comparison of demographic and clinical variables between successful and unsuccessful scans in autism spectrum condition and typically developing control groups (grouping 2) Table 3 long description.

Variable	ASC-II (*n* = 46)		ASC-IA (*n* = 37)		TDC (*n* = 39)	
	Successful (*n* = 22)	Statistics	Successful (*n* = 24)	Statistics	Successful (*n* = 33)	Statistics
	Not successful (*n* = 24)		Not successful (*n* = 13)		Not successful (*n* = 6)	
Demographics						
Age, years	18 [13]	*U* = 141, *q* = 0.275	17.5 [8]	*U* = 59, *q* = 0.022	18 [7]	*U* = 13.5, *q* < 0.001
	14 [9]		10 [3]		9.5 [3]	
ASC profile						
ADOS-2 CSS^a^	7 [3]	*U* = 189.5, *q* = 0.969	5 [3]	*U* = 130, *q* = 0.590	—	
	6 [3]		5 [3]			
ADI-R total	23 [7]	*U* = 141, *q* = 0.275	20 [8]	*U* = 111, *q* = 0.590	—	
	25 [7]		21.5 [9]			
SRS total	96 [32]	*U* = 189, *q* = 0.969	86 [38]	*U* = 127, *q* = 0.929	18 [13]	*U* = 73, *q* = 0.346
	97 [33]		83.5 [38]		21 [17]	
SSP total	27.5 [44]	*U* = 183, *q* = 0.969	26 [22]	*U* = 91.5, *q* = 0.280	3 [8]	*U* = 98.5, *q* = 0.899
	35.5 [23]		40 [31]		2 [17]	
RBS-R total	17.5 [44]	*U* = 186.5, *q* = 0.969	15 [29]	*U* = 87.5, *q* = 0.280	1 [4]	*U* = 56.5, *q* = 0.448
	25 [24]		28 [20]		3.5 [9]	
Functional profile						
FIQ^b^	76 [15]	*U* = 74.5, *q* = 0.226	104 [18]	*U* = 149, *q* = 0.928	113 [15]	*U* = 88, *q* = 0.782
	66 [25]		99 [27]		110 [22]	
Leiter-R NVFIQ	90 [26]	*U* = 127, *q* = 0.275	120.5 [25]	*U* = 121, *q* = 0.280	123 [12]	*U* = 50.5, *q* = 0.021
	67 [50]		111 [20]		110 [21]	
VABS ABC	68.5 [10]	*U* = 117, *q* = 0.226	90.5 [20]	*U* = 131.5, *q* = 0.907	113 [32]	*U* = 61, *q* = 0.448
	61 [12]		89 [24]		106 [31]	
BRIEF-GEC	153 [25]	*U* = 189, *q* = 0.969	154 [39]	*U* = 89.5, *q* = 0.310	84 [17]	*U* = 18, *q* < 0.001
	163 [49]		164 [34]		116.5 [27]	
Behavioural profile						
ABC total	43 [57]	*U* = 190.5, *q* = 0.969	35 [41]	*U* = 113.5, *q* = 0.590	0 [3]	*U* = 42.5, *q* = 0.340
	55.5 [65]		31.5 [49]		7.5 [13]	
SNAP-IV total	19 [12]	*U* = 133, *q* = 0.314	20.5 [12]	*U* = 80.5, *q* = 0.280	2 [6]	*U* = 16, *q* < 0.001
	25 [18]		27.5 [22]		12 [5]	

Scans were successful if participant completed sMRI, rsfMRI, and dMRI. All continuous variables are presented as median [interquartile range].

ASC-II, autism spectrum condition with intellectual impairment only or autism spectrum condition with intellectual impairment and minimally verbal status; ASC-IA, intellectually able autism spectrum condition; TDC, typically developing controls; ASC, autism spectrum condition; ADOS-2 CSS, Autism Diagnostic Observation Schedule-Second Edition, calibrated severity score; ADI-R, Autism Diagnostic Interview-Revised, total score based on the current algorithm; SRS, Social Responsiveness Scale; SSP, Short Sensory Profile; RBS-R, Repetitive Behavior Scale-Revised; FIQ, Wechsler Intelligence Scale, Full-scale Intelligence Quotient; Leiter-R NVFIQ, Leiter International Performance Scale-Revised, Non-verbal Full Intelligence Quotient; VABS ABC, Vineland Adaptive Behavior Scale, Adaptive Behavior Composite; BRIEF-GEC, Behavior Rating Inventory of Executive Function, Global Executive Composite; ABC, Aberrant Behavior Checklist; SNAP-IV, Swanson, Nolan, and Pelham-IV Questionnaire; MRI, magnetic resonance imaging; sMRI, structural MRI (T1-weighted image); rsfMRI, resting-state functional MRI; dMRI, diffusion MRI.

a. Mann–Whitney *U*-test was used followed by the Benjamini–Hochberg procedure to give false discovery rate-adjusted *p*-values (*q*-values), calculated separately for each diagnostic categories.

b. Wechsler Adult Intelligence Scale, Fourth Edition was used for participants aged 16 years and above; Wechsler Intelligence Scale for Children, Fourth Edition was used for participants younger than 16 years.

**Table 4:** Spearman rank correlation between demographic or clinical variables and quality control metrics for MRI scans Table 4 long description.

Variable	sMRI (IQR)	rsfMRI (mean framewise displacement)	dMRI (relative root-mean-square)
Demographics			
Age	*rho* = 0.194, *q* = 0.049	*rho* = −0.450, *q* < 0.001	*rho* = −0.220, *q* = 0.037
ASC profile			
ADOS-2 CSS^a^	*rho* = −0.221, *q* = 0.086	*rho* = 0.169, *q* = 0.205	*rho* = 0.310, *q* = 0.018
ADI-R total^a^	*rho* = −0.104, *q* = 0.401	*rho* = 0.146, *q* = 0.267	*rho* = 0.062, *q* = 0.620
SRS total	*rho* = −0.362, *q* < 0.001	*rho* = 0.322, *q* = 0.002	*rho* = 0.109, *q* = 0.292
SSP total	*rho* = −0.405, *q* < 0.001	*rho* = 0.315, *q* = 0.003	*rho* = 0.120, *q* = 0.259
RBS-R total	*rho* = −0.383, *q* < 0.001	*rho* = 0.347, *q* = 0.001	*rho* = 0.148, *q* = 0.174
Functional profile			
FIQ^b^	*rho* = 0.307, *q* = 0.003	*rho* = −0.331, *q* = 0.002	*rho* = −0.152, *q* = 0.167
Leiter-R NVFIQ	*rho* = 0.326, *q* = 0.001	*rho* = −0.347, *q* < 0.001	*rho* = −0.245, *q* = 0.019
VABS ABC	*rho* = 0.388, *q* < 0.001	*rho* = −0.383, *q* < 0.001	*rho* = −0.168, *q* = 0.120
BRIEF-GEC	*rho* = −0.465, *q* < 0.001	*rho* = 0.376, *q* < 0.001	*rho* = 0.194, *q* = 0.074
Behavioural profile			
ABC total	*rho* = −0.431, *q* < 0.001	*rho* = 0.308, *q* = 0.002	*rho* = 0.132, *q* = 0.216
SNAP-IV total	*rho* = −0.483, *q* < 0.001	*rho* = 0.405, *q* < 0.001	*rho* = 0.222, *q* = 0.042

Correlation coefficient (*rho*) reported. False discovery rate correction: The *q*-values were calculated using the Benjamini–Hochberg procedure for 36 pairs (12 demographic/clinical variables × 3 quality control metrics) of Spearman rank correlation.

MRI, magnetic resonance imaging; sMRI, structural MRI (T1-weighted image); IQR, CAT12’s image quality rating; rsfMRI, resting-state functional MRI; dMRI, diffusion MRI; ASC, autism spectrum condition; ADOS-2 CSS, Autism Diagnostic Observation Schedule-Second Edition, calibrated severity score; ADI-R, Autism Diagnostic Interview-Revised, total score based on the current algorithm; SRS, Social Responsiveness Scale; SSP, Short Sensory Profile; RBS-R, Repetitive Behavior Scale-Revised; FIQ, Wechsler Intelligence Scale, Full-scale Intelligence Quotient; Leiter-R NVFIQ, Leiter International Performance Scale-Revised, Non-verbal Full Intelligence Quotient; VABS ABC, Vineland Adaptive Behavior Scale, Adaptive Behavior Composite; BRIEF-GEC, Behavior Rating Inventory of Executive Function, Global Executive Composite; ABC, Aberrant Behavior Checklist; SNAP-IV, Swanson, Nolan, and Pelham-IV Questionnaire.

a. Data were not collected for typically developing controls.

b. Wechsler Adult Intelligence Scale, Fourth Edition was used for participants aged 16 years and above; Wechsler Intelligence Scale for Children, Fourth Edition was used for participants younger than 16 years.

## Results

### Participant characteristics

Demographic and clinical characteristics of participants are detailed by diagnostic subgroupings. When comparing groups ASC-IA, ASC-II and TDCs (grouping 2; Table 1), the three groups were similar in age and gender ratio. As anticipated, both ASC-IA and ASC-II groups presented with more co-occurring conditions and higher rates of medication use compared with the TDC group. Within the autistic participants, the ASC-II subgroup showed modestly but significantly higher researcher-rated ADOS-2 CSS and ADI-R total scores than the ASC-IA subgroup. However, parent ratings of autistic symptoms, executive dysfunction, behavioural problems and ADHD symptoms were comparable between the ASC-IA and ASC-II subgroups. Significant differences in cognitive functioning were observed, with the ASC-II group demonstrating lower scores on the Wechsler FIQ, Leiter-R Non-Verbal Full Intelligence Quotient (Leiter-R NVFIQ), and VABS ABC compared with the ASC-IA group. Further analysis comparing all three autistic subgroups (ASC-IA, ASC-IIO and ASC-MV) with TDCs (grouping 3; Supplementary Table 3) revealed that all autistic subgroups had lower Wechsler FIQ, Leiter-R NVFIQ and VABS ABC scores, and higher BRIEF-GEC scores than TDCs. Additionally, all autistic groups exhibited more severe behavioural problems (ABC total scores) and ADHD symptoms (SNAP-IV total scores) than the TDC group. Regarding MRI data quality, the ASC group exhibited significantly lower sMRI quality (higher IQR; *p* < 0.001) and increased rsfMRI head motion (higher mean framewise displacement; *p* = 0.002) compared with TDCs, whereas no significant group differences were found for dMRI motion or rsfMRI ME-ICA component ratios (Table 1). Subgroup analyses confirmed that sMRI quality reductions were pervasive across all ASC profiles (*p* < 0.001), whereas elevated rsfMRI motion was driven largely by the ASC-MV and ASC-II subgroups (Supplementary Tables 2 and 3).

### Success rates by group

**Fig. 2:**
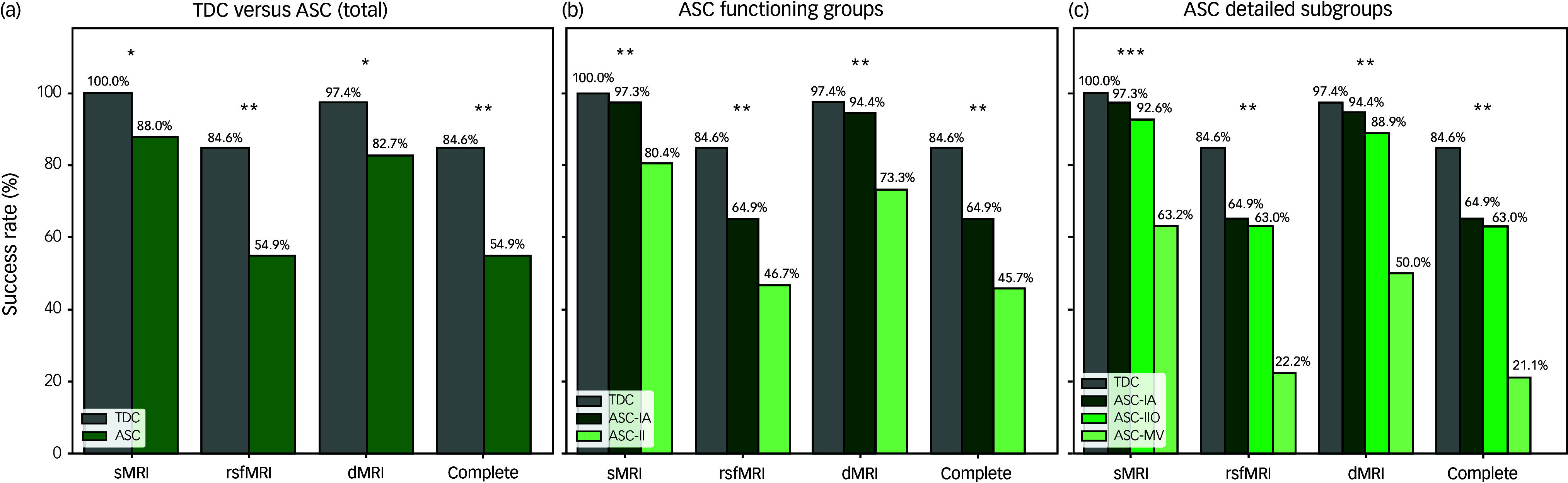
Fig. 2 long description. Magnetic resonance imaging (MRI) scan success rates by scan modality and diagnostic subgroup. Bar charts display the percentage of participants who successfully completed usable scans for structural MRI (sMRI), resting-state functional MRI (rsfMRI), diffusion MRI (dMRI) and the complete protocol (all three modalities). (a) Comparison between the total autism spectrum condition (ASC) cohort and typically developing controls (TDCs). (b) Comparison based on intellectual functioning: TDC, intellectually able ASC (ASC-IA) and ASC with intellectual impairment (ASC-II). (c) Detailed subgroup breakdown: ASC-IA, ASC with intellectual impairment only (ASC-IIO) and ASC with minimally verbal status (ASC-MV). Significant group differences were determined using chi-squared tests with false discovery rate correction (Benjamini–Hochberg procedure calculated for all 12 comparison conditions). Significance levels are indicated as: *0.01 < *q* < 0.05, ** 0.001 < *q* < 0.01, *** *q* < 0.001.

Table 2 and Fig. 2 present the MRI success rates across the three defined participant groupings for sMRI, rsfMRI and dMRI, as well as for completion of all three sequences. Overall, as shown in Fig. 2(a) (grouping 1), the combined ASC group had significantly lower success rates compared with the TDC group for all modalities. When the ASC group was subdivided by intellectual capacity (Fig. 2(b); grouping 2), the ASC-II subgroup consistently showed lower success rates than both the ASC-IA subgroup and TDC group across all scan types. In contrast, success rates for the ASC-IA group did not differ significantly from controls.

Crucially, further subdivision of the ASC-II group revealed that the ASC-MV subgroup had the lowest success rates of all groups (Fig. 2(c); grouping 3). The ASC-MV group exhibited significantly lower completion rates compared with the ASC-IIO, ASC-IA and TDC groups across sMRI, rsfMRI and dMRI sequences. This highlights the particular challenges in acquiring usable MRI data from autistic individuals with co-occurring intellectual impairment and minimal verbal abilities.

### Characteristics of successful and unsuccessful scans/participants

Demographic and clinical characteristics differentiating participants with successful versus unsuccessful completion of all MRI sequences are presented in Table 3 and Supplementary Tables 4 and 5. Within the TDC group (grouping 1; Table 3), successful participants were significantly older (*q* < 0.001) and demonstrated higher non-verbal FIQ (*q* = 0.021), better executive function (*q* < 0.001) and fewer ADHD symptoms (*q* < 0.001), compared with those who did not successfully complete the MRI procedure. Among autistic participants (grouping 1; Supplementary Table 4), those who successfully completed all MRI sequences were also significantly older (*q* = 0.010), had lower ADI-R total scores (*q* = 0.043), fewer ADHD symptoms (*q* = 0.048), higher non-verbal FIQ (*q* = 0.017) and better adaptive function (*q* = 0.032). However, when autistic participants were further subgrouped (Table 3 and Supplementary Table 5), many of these differentiating trends were no longer significant, with age being the only variable that remained significantly different between successful and unsuccessful participants within the ASC-IA subgroup (*q* = 0.022).

### Dimensional relationships between clinical characteristics and data quality

**Fig. 3:**
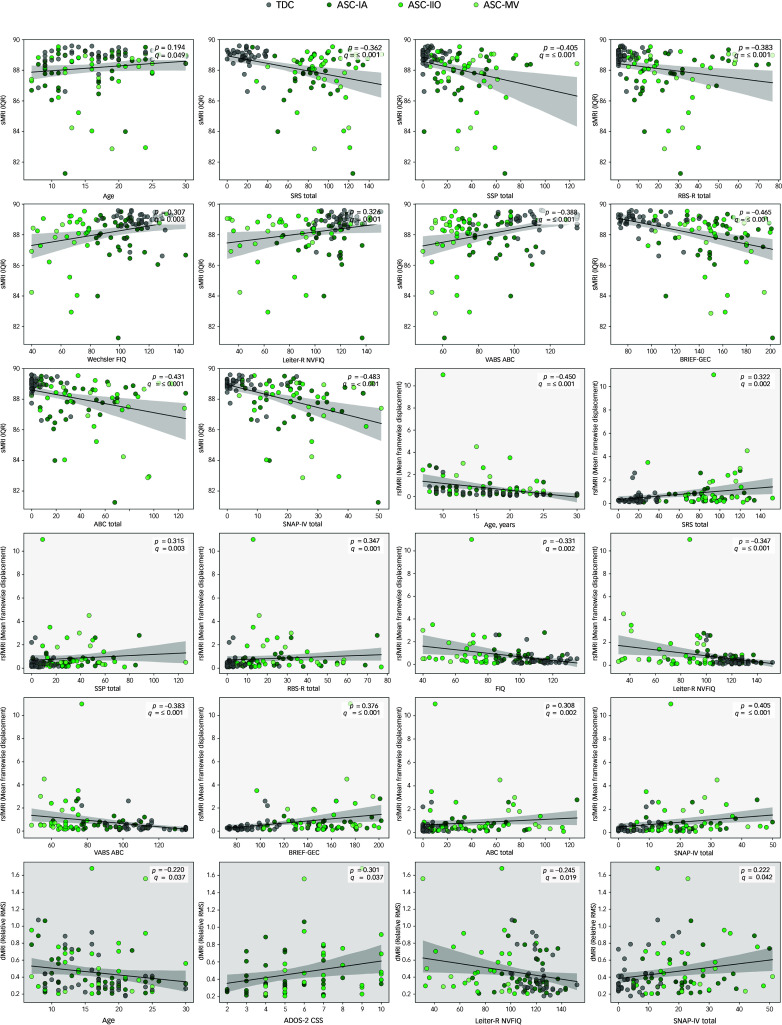
Fig. 3 long description. Associations between demographic or clinical profiles and MRI quality metrics. Scatterplots depicting significant Spearman rank correlations (*q* < 0.05) between participant characteristics (*x*-axis) and quality control metrics (*y*-axis) for sMRI (CAT12 IQR), rsfMRI (mean framewise displacement) and dMRI (mean relative root-mean-square). Panels are organised by imaging modality, with background shading used to distinguish scan types: white for sMRI (CAT12 IQR), very light grey for rsfMRI (mean framewise displacement) and light grey for dMRI (mean relative root-mean-square). Each data point represents a single participant, colour-coded by subgroup: TDCs (grey), ASC-IA (dark green), ASC-IIO (lime green) and ASC-MV (pale green). The solid black line indicates the overall linear regression trend, with the shaded region representing the 95% confidence interval. The *rho* value denotes Spearman’s rank correlation coefficient. The *q*-value denotes the false discovery rate-corrected *p*-value, calculated using the Benjamini–Hochberg procedure for 36 pairs (12 demographic/clinical variables × 3 quality control metrics) of correlation. Correlations for the ADOS-2 CSS and ADI-R were performed within the ASC cohort only. ABC, Aberrant Behavior Checklist; ADOS-2 CSS, Autism Diagnostic Observation Schedule-Second Edition, calibrated severity score; ASC, autism spectrum condition; ASC-IA, intellectually able ASC; ASC-II, ASC with intellectual impairment (combined ASC-IIO and ASC-MV); ASC-IIO, ASC with intellectual impairment only; ASC-MV, ASC with minimally verbal status; BRIEF-GEC, Global Executive Composite of the Behavior Rating Inventory of Executive Function; dMRI, diffusion MRI; FIQ, Wechsler Full-Scale Intelligence Quotient; IQR, image quality rating; Leiter-R NVFIQ, Leiter International Performance Scale – Revised, Non-Verbal Full Intelligence Quotient; MRI, magnetic resonance imaging; RBS-R, Repetitive Behavior Scale – Revised; RMS, root-mean-square; rsfMRI, resting-state functional MRI; sMRI, structural MRI (T1-weighted); SNAP-IV, Swanson, Nolan, and Pelham-IV Questionnaire; SSP, Short Sensory Profile; TDCs, typically developing controls; VABS ABC, Adaptive Behavior Composite of the Vineland Adaptive Behavior Scales.

We further examined the dimensional relationships between participant characteristics and continuous metrics of image quality (sMRI IQR, rsfMRI mean framewise displacement, dMRI relative root-mean-square) (Table 4). Figure 3 visualises the significant correlations between clinical variables and data quality.

For sMRI, higher IQR was associated with older age, higher cognitive and adaptive functioning (FIQ, VABS ABC), and lower severity of autistic symptoms (SRS, SSP, RBS-R), behavioural problems (ABC) and ADHD symptoms (SNAP-IV).

For rsfMRI, lower head motion (mean framewise displacement, where lower indicates better quality/less motion) was significantly correlated with older age and lower severity of autistic and ADHD symptoms. Interestingly, although behavioural problems (ABC scores) were strongly linked to motion, overall cognitive scores (FIQ) showed a weaker association with fMRI motion compared with sMRI quality.

For dMRI, head motion (mean relative root-mean-square, where lower indicates better quality/less motion) showed weaker correlations with clinical variables compared with other modalities, although significant associations were still observed with age and specific symptom severities (Fig. 3).

These comprehensive correlational analyses (Table 4) fulfil a key aim of this study by demonstrating that beyond diagnostic categories, specific demographic (age), cognitive (e.g. non-verbal IQ, adaptive functioning) and behavioural characteristics (e.g. autistic symptom severity, ADHD symptoms, executive function) are significantly linked to the quality of multi-modal MRI data.

## Discussion

A pivotal challenge in autism neuroimaging is the frequent exclusion of individuals with co-occurring intellectual impairment and/or minimally verbal status. Our study makes a significant contribution by not only successfully including these individuals, but also systematically identifying factors that predict MRI scan success and data quality across a diverse autistic cohort. This approach is crucial for developing more equitable research practices and generating findings that are truly representative of the autism spectrum. By adopting a binary ‘pass/fail’ criterion for scan success and quantitative measures of image data quality, we aimed to provide a nuanced understanding of factors influencing MRI data acquisition quality in this heterogeneous population.

Consistent with previous work,^15,17^ age emerged as a significant predictor of scan success. This highlights the ongoing need to tailor neuroimaging protocols for younger participants, considering developmental factors like attention span and comfort with the scanning environment.^54^ Future research should continue to explore age-specific interventions, such as age-appropriate explanations and optimised scan durations.

Our comprehensive support protocol^13,15,16,54^ was critical to our success in recruiting and scanning a diverse sample. Indeed, our overall scan success rates, even when including participants with intellectual impairment and minimally verbal status, were comparable with previous literature that employed intensive support strategies.^15,16^ A necessary trade-off of this ‘bundled’ support protocol (mock scanner, video modelling, Inscapes) is that it precludes the statistical isolation of each strategy’s independent efficacy. However, utilisation patterns of the optional components offer insights into their relative utility. Namely, the mock scanner was utilised predominantly by participants in the ASC-MV (*n* = 13) and ASC-IIO (*n* = 8) subgroups, compared with only four participants in the ASC-IA group. This trend suggests that physical desensitisation may be a ‘high-yield’ strategy for autistic individuals with lower cognitive or verbal abilities, whereas video modelling may be sufficient for many individuals with intact cognitive functioning. Although we did not collect systematic quantitative data on caregiver satisfaction, a limitation of the current study design, qualitative feedback indicated a strong preference for this flexible approach, with families noting that the ability to tailor the intensity of preparation (e.g. opting in or out of the mock scan) reduced burden while maintaining support.

Our experience in this study, consistent with previous reports,^15^ suggests that mock scanner sessions serve as a valuable tool for autistic participants to acclimate to the scanning environment, which has previously been shown to significantly improve MRI completion rates in autistic individuals with intellectual impairment.^15^ Additionally, the presence of an acquainted caregiver or therapist during the scan provides emotional support and reassurance, which is crucial for autistic participants as they tend to have high anxiety levels in unfamiliar environments. Familiarisation protocols, such as exposure to the MRI environment through videos or sound recordings before the actual scan, can also help reduce sensory sensitivities and prepare participants for the scanning experience. The success rates for sMRI and dMRI scans were especially good, suggesting that using the Inscapes movie paradigm may play a key role in these outcomes. Inscapes, a visually engaging yet non-social movie, helps maintain participant engagement and reduce motion by providing a calming distraction without requiring active cognitive engagement.^39^ Incorporating such stimuli may help minimise head motion, thereby improving data quality, particularly during longer sessions. However, a key finding was that autistic individuals with co-occurring minimally verbal status (the ASC-MV group) had the lowest scan success rates across all MRI sequences. This, despite our extensive support measures, underscores the profound challenges in acquiring usable data from this segment of the autistic population, and signals an urgent need for further research into innovative, highly individualised and potentially more resource-intensive strategies for this specific subgroup.

It is important to contextualise these findings regarding behavioural support against the alternative of sedation. Although sedation is frequently used to ensure scan completion in individuals with intellectual impairment, it carries inherent medical risks and logistical burdens.^55^ Importantly, anaesthetic agents can alter BOLD signal properties and functional connectivity patterns,^56^ limiting the ecological validity of the data. Our study demonstrates that with comprehensive behavioural support and advanced acquisition sequences, high-quality data can be acquired in complex populations without the confounds of sedation.^13^

Our study also demonstrated the benefits of specific advanced imaging techniques; the use of multiband multi-echo imaging sequences not only reduced scan times – thereby lessening participant burden and potential anxiety – but also holds promise for improving data quality by mitigating motion artifacts. Specifically, the use of multiband imaging sequence acceleration enabled quicker scanning (sMRI: around 5 min versus around 7 min using the legacy magnetisation prepared rapid gradient echo imaging sequence; dMRI: around 8 min versus around 18 min using the legacy high angular resolution diffusion imaging sequence). Further, the adoption of multi-echo EPI sequences, which allowed for the optimised separation of BOLD signal from non-BOLD components, enhances fMRI data quality.^45^ This can help mitigate the impact of head motion, ultimately increasing the number of usable data-sets, improving the robustness of findings and reducing data loss, which are always the methodological concerns for imaging neuroscience in autistic people.^2^ Furthermore, our analysis of the fMRI data quality suggests that the use of multi-echo denoising (ME-ICA) is a robust strategy for inclusive research. Although autistic participants – particularly those with intellectual impairment – exhibited significantly higher head motion (framewise displacement) than controls, the ratio of accepted BOLD-like components to rejected artifact components remained comparable across all groups. This stability suggests that the multi-echo denoising algorithm was equally effective at separating neural signal from motion artifacts across the functioning spectrum. By effectively managing the artifacts inherent in scanning lower-functioning individuals, these techniques ensure that the retained data preserves robust physiological signal even in the presence of increased motion. This benefit from multi-echo techniques endures even when some fMRI quality metrics exceed common cut-offs (e.g. mean framewise displacement > 0.5 mm).^43–45^ These technical advancements are particularly pertinent when working with autistic individuals who may have limited tolerance for prolonged scans,^37,57^ including those with intellectual impairment and minimally verbal status,^9^ and are crucial for improving the feasibility and quality of inclusive neuroimaging research.

Notably, we prioritised framewise displacement and the ME-ICA component acceptance ratio as our primary quality metrics because they directly index participant adherence^49^ and algorithmic stability.^43–45^ We did not rely on signal-variance metrics such as derivative variance over voxels or temporal signal-to-noise ratio^58^ because the synthetic reconstruction of time-series data in ME-ICA alters signal properties, rendering standard single-echo benchmarks less interpretable.^43–45^ Furthermore, a direct statistical comparison between multi-echo and legacy single-echo pipeline would quantify the specific incremental benefit of this sequence,^59^ but such a methodological validation was outside the current study’s scope. Although ME-ICA/tedana produces component-classification summaries (accepted/rejected/ignored), there is currently no consensus threshold for an ‘acceptable’ acceptance fraction because component counts and variance partitions depend on acquisition parameters and denoising settings.^43–45^ Therefore, we did not use component retention ratios as inclusion/exclusion criteria.

A major contribution of this study is the detailed examination of how clinical, cognitive and behavioural characteristics relate to quantitative MRI image quality metrics (Table 4). We found that higher levels of autistic symptoms, increased ADHD symptoms, behavioural challenges, executive dysfunction, and lower cognitive and adaptive functioning were significantly associated with poorer image quality.^15,16,54,60^ This is particularly noteworthy as many neuroimaging studies focus primarily on scan success rates rather than these nuanced relationships between participant characteristics and data quality. Understanding these links is vital for developing more tailored support procedures and refined imaging protocols.

The identification of factors such as age, ADHD symptoms and non-verbal intelligence as key predictors of MRI success has direct clinical relevance. Psychiatrists referring autistic patients for clinical MRI scans can use this knowledge to anticipate potential challenges, advocate for appropriate support measures (e.g. familiarisation protocols, presence of caregiver, quieter sequences if available) and better prepare patients and families, potentially improving the diagnostic yield and patient experience. Furthermore, the association between ADHD symptoms and lower data quality highlights the importance of considering and managing co-occurring conditions when planning any procedure requiring sustained attention and stillness in autistic individuals. These insights extend beyond autism, as many individuals with other neurodevelopmental or psychiatric conditions may face similar challenges during medical procedures.

The primary strengths of this study include the recruitment of a deeply phenotyped and diverse sample of autistic individuals, with significant representation of those with intellectual impairment and minimally verbal status – populations often excluded from neuroimaging research. Additionally, the implementation of a comprehensive, multifaceted support protocol and the use of both binary and quantitative outcome measures for scan success and data quality enhance the robustness of our findings. Despite these strengths, several limitations warrant discussion. First, data were acquired at a single site, limiting generalisability. Second, the cross-sectional design prevents causal inferences regarding the relationship between age and scan success. Third, regarding medication status, we employed a standard 24 h washout period to minimise acute pharmacological effects. However, we acknowledge that this duration may be insufficient to eliminate the neurophysiological adaptations associated with chronic stimulant treatment. Recent work suggests that long-term psychostimulant use may induce lasting neurophysiological effects.^61^ Fourth, our sample was predominantly male, which may limit generalisability to autistic females who can present with different phenotypes.^62^ Fifth, the presence of co-occurring psychiatric conditions and psychotropic medication use, while reflecting real-world clinical complexity, may introduce confounds. Sixth, the wide age range (7–30 years) was necessary to facilitate recruitment of participants with significant support needs and ensure a representative sample of the autism spectrum. However, this breadth implies that developmental trajectories or learned adaptations might have introduced residual variability into the data. Future research should consider age-stratified designs to better isolate these developmental factors. Seventh, although our sample size is substantial for a study of this nature, particularly given the targeted recruitment of individuals with intellectual impairment/minimally verbal status, the sample size of the minimally verbal subgroup remained relatively small, limiting the power for subgroup-specific analyses and for the detection of more subtle effects. Finally, the absence of a control group with intellectual disability without autism means we cannot fully disentangle the specific contributions of autism versus intellectual disability to MRI scan outcomes.

In conclusion, this study underscores the critical importance of individualised and supportive approaches to facilitate successful and high-quality MRI data acquisition in autistic individuals, especially those with co-occurring intellectual impairment or minimally verbal status. The deliberate inclusion of these participants is paramount if neuroimaging research is to accurately reflect the full spectrum of autism and generate findings that benefit all autistic people. Our results demonstrate that specific participant characteristics significantly affect scan success and image quality. These findings pave the way for more inclusive neuroimaging methodologies and provide actionable insights for both research and clinical settings. Ultimately, advancing our understanding of autism and other neurodevelopmental conditions requires a commitment to research that mirrors the true heterogeneity of these populations. This study offers empirical evidence for factors that can facilitate more inclusive and successful neuroimaging, not only for autistic individuals with significant support needs, but potentially for other clinical populations facing similar procedural challenges. Adopting such tailored and supportive approaches is essential for both ethical research and effective clinical care in psychiatry.

## Supporting information

10.1192/bjo.2026.12035.sm001 Huang et al. supplementary material Huang et al. supplementary material

## Data Availability

The data that support the findings of this study are available on request from the corresponding author, H.-Y.L.

## Footnotes

a. We use the term ‘autism spectrum condition’ (ASC) to reflect a neurodiversity-oriented perspective, acknowledging that ‘autism spectrum disorder’ (ASD) is the standard diagnostic terminology used in the DSM-5.

## Supplementary material

The supplementary material is available online at https://doi.org/10.1192/bjo.2026.12035

## Fig. 1 Long description

The flowchart begins with 125 recruited participants. Three participants are excluded because they did not attempt the scan. The remaining participants are divided into two main groups: 83 with autism spectrum condition (ASC) and 39 typically developing controls (TDC). The ASC group is further divided into intellectually able (ASC-IA) with 37 participants who have a full-scale intelligence quotient (FIQ) of 85 or higher and an adaptive behavior composite (VABS ABC) of 85 or higher. The other subgroup within ASC is those with intellectual impairment (ASC-II) totaling 46 participants, who have an FIQ of less than 85 or a VABS ABC of less than 85. The ASC-II group is further split into two: those with intellectual impairment only (ASC-IIO) with 27 participants and those who are minimally verbal (ASC-MV) with 19 participants.

Navigate back to Fig. 1.

## Table 1 Long description

The table presents a comparison of demographic and clinical profiles of participants across three groups: ASC-II with forty-six participants, ASC-IA with thirty-seven participants, and TDCs with thirty-nine participants. The table includes data on age, gender, medication, and comorbidity. It also provides ASC profiles such as ADOS-2 CSS, ADI-R total, SRS total, SSP total, and RBS-R total. Functional profiles include FIQ, Letter-R NVFIQ, VABS ABC, BRIEF-GEC, and SNAP-IV total. Behavioral profiles include ABC total and SNAP-IV total. MRI quality metrics include sMR, fMR, dMR, and tMR. The table provides statistical analysis and post hoc comparisons between the groups.

Navigate back to Table 1.

## Table 2 Long description

The table presents a comparison of MRI success rates by scan type between various subgroupings of participants with autism spectrum condition and typically developing controls. It includes data for sMRI, rsfMRI, dMRI, and the completion of all three scans. The table has four main sections, each representing different groupings of participants. Each section lists the success rates, statistics, and post hoc results for each subgroup. For example, in Grouping 1, the ASC subgroup shows a success rate of seventy-three successful out of one hundred for sMRI, while the TDC subgroup shows a success rate of thirty-nine successful out of one hundred. The table also includes statistical values and post hoc comparisons, indicating significant differences between subgroups. The data highlights variations in MRI success rates across different scan types and participant groupings.

Navigate back to Table 2.

## Table 3 Long description

The table presents a comparison of demographic and clinical variables between successful and unsuccessful scans in autism spectrum condition (ASC) and typically developing control (TDC) groups. It is divided into three main sections: ASC-II, ASC-IA, and TDC, each with columns for successful and not successful scans. The table includes statistics for various measures such as age, ADOS-2 CSS, ADI-R total, SRS total, SSP total, RBS-R total, FIQ, Leiter-R NVFIQ, VABS ABC, BREF-GEC, ABC total, SNAP-IV total, and more. Each section provides median values and U statistics with q values for non-parametric tests. Notable trends include significant differences in age between successful and not successful scans in the ASC-IA group and various clinical measures across groups. The table highlights the use of non-parametric statistics due to violations of the normality assumption.

Navigate back to Table 3.

## Table 4 Long description

The table presents Spearman rank correlation coefficients between demographic or clinical variables and quality control metrics for structural MRI (sMRI), resting-state functional MRI (rsfMRI), and diffusion MRI (dMRI). It includes correlations for age, ASC profile, functional profile, and behavioral profile across three MRI quality metrics. The table has 15 rows and 4 columns, with columns labeled Demographics, sMRI (IQR), rsfMRI (mean framewise displacement), and dMRI (relative root-mean-square). Each row lists a variable, its correlation coefficient (rho), and significance value (q) for each MRI metric. Notable trends include significant correlations for SRS total, FIQ, and ABC total with all MRI metrics, indicating strong relationships between these clinical variables and MRI quality.

Navigate back to Table 4.

## Fig. 2 Long description

The bar graph compares MRI scan success rates by scan modality and diagnostic subgroup, displaying the percentage of participants who successfully completed usable scans for structural MRI (sMRI), resting-state functional MRI (rsfMRI), diffusion MRI (dMRI), and the complete protocol (all three modalities). The graph is divided into three panels. Panel A compares the total autism spectrum condition (ASC) cohort with typically developing controls (TDCs). Panel B compares groups based on intellectual functioning: TDC, intellectually able ASC (ASC-IA), and ASC with intellectual impairment (ASC-II). Panel C provides a detailed subgroup breakdown: ASC-IA, ASC with intellectual impairment only (ASC-IIO), and ASC with minimally verbal status (ASC-MV). The x-axis lists the scan modalities (sMRI, rsfMRI, dMRI, Complete), and the y-axis shows the success rate in percentage. The bars are color-coded: gray for TDC, dark green for ASC, and light green for ASC-II. Significant group differences are indicated with asterisks: one asterisk for 0.01 < q < 0.05, two asterisks for 0.001 < q < 0.01, and three asterisks for q < 0.001. Notable trends include higher success rates for TDCs across all modalities compared to ASC groups. The ASC-II group shows the lowest success rates, particularly in the complete protocol. All values are approximated.

Navigate back to Fig. 2.

## Fig. 3 Long description

The image contains multiple scatterplots depicting significant Spearman rank correlations between participant characteristics and MRI quality metrics. The x-axis represents various participant characteristics such as age, SRS total, SSP total, RBS-R total, Wechsler FIQ, Leiter-R NVFIQ, VABS ABC, BRIEF-GEC, ASC total, SNAP-IV total, ADOS-2 CSS, and SNAP-IV total. The y-axis represents quality control metrics for sMRI (CAT12 IQR), rsfMRI (mean framewise displacement), and dMRI (mean relative root-mean-square). Each data point represents a single participant, color-coded by subgroup: TDCs (grey), ASC-IA (dark green), ASC-IIO (lime green), and ASC-MV (pale green). The solid black line indicates the overall linear regression trend, with the shaded region representing the 95% confidence interval. The rho value denotes Spearman’s rank correlation coefficient, and the q-value denotes the false discovery rate-corrected p-value. Correlations for the ADOS-2 CSS and ADI-R were performed within the ASC cohort only. The scatterplots are organized by imaging modality, with background shading used to distinguish scan types: white for sMRI, very light grey for rsfMRI, and light grey for dMRI. All values are approximated.

Navigate back to Fig. 3.

## References

[1] Kenny L , Hattersley C , Molins B , Buckley C , Povey C , Pellicano E. Which terms should be used to describe autism? Perspectives from the UK autism community. Autism 2016; 20: 442–62. 26134030 10.1177/1362361315588200

[2] Lin H-Y , Lai M-C. The neuroradiology of autism: framing neuroimaging investigations of the autistic brain based on the US NIMH Research Domain Criteria. In Neurodevelopmental Pediatrics: Genetic and Environmental Influences (eds DD Eisenstat, D Goldowitz , TF Oberlander , JY Yager ): 269–82. Springer International Publishing, 2023.

[3] Mash LE , Reiter MA , Linke AC , Townsend J , Müller RA. Multimodal approaches to functional connectivity in autism spectrum disorders: an integrative perspective. Dev Neurobiol 2018; 78: 456–73. 29266810 10.1002/dneu.22570 PMC5897150

[4] Wang M , Xu D , Zhang L , Jiang H. Application of multimodal MRI in the early diagnosis of autism spectrum disorders: a review. Diagnostics (Basel) 2023; 13: 3027. 37835770 10.3390/diagnostics13193027 PMC10571992

[5] Tager-Flusberg H , Kasari C. Minimally verbal school-aged children with autism spectrum disorder: the neglected end of the spectrum. Autism 2013; 6: 468–78. 10.1002/aur.1329 PMC3869868 24124067

[6] Posar A , Visconti P. Update about ‘minimally verbal’ children with autism spectrum disorder. Rev Paul Pediatr 2021; 40: e2020158. 34495269 10.1590/1984-0462/2022/40/2020158 PMC8432069

[7] Rose V , Trembath D , Keen D , Paynter J. The proportion of minimally verbal children with autism spectrum disorder in a community-based early intervention programme. J Intellect Disabil Res 2016; 60: 464–77. 27120989 10.1111/jir.12284

[8] Maenner MJ , Warren Z , Williams AR , Amoakohene E , Bakian AV , Bilder DA , et al. Prevalence and characteristics of autism spectrum disorder among children aged 8 years – autism and developmental disabilities monitoring network, 11 sites, United States, 2020. MMWR Surveill Summ 2020; 72: 1–14. 10.15585/mmwr.ss7202a1 PMC10042614 36952288

[9] Jack A , Pelphrey AK. Annual research review: understudied populations within the autism spectrum - current trends and future directions in neuroimaging research. J Child Psychol Psychiatry 2017; 58: 411–35. 28102566 10.1111/jcpp.12687 PMC5367938

[10] Lord C , Brugha TS , Charman T , Cusack J , Dumas G , Frazier T , et al. Autism spectrum disorder. Nat Rev Dis Primers 2020; 6: 5. 31949163 10.1038/s41572-019-0138-4 PMC8900942

[11] Zeidan J , Fombonne E , Scorah J , Ibrahim A , Durkin MS , Saxena S , et al. Global prevalence of autism: a systematic review update. Autism Res 2022; 15: 778–90. 35238171 10.1002/aur.2696 PMC9310578

[12] Lombardo MV , Lai MC , Baron-Cohen S. Big data approaches to decomposing heterogeneity across the autism spectrum. Mol Psychiatry 2019; 24: 1435–50. 30617272 10.1038/s41380-018-0321-0 PMC6754748

[13] Stogiannos N , Carlier S , Harvey-Lloyd JM , Brammer A , Nugent B , Cleaver K , et al. A systematic review of person-centred adjustments to facilitate magnetic resonance imaging for autistic patients without the use of sedation or anaesthesia. Autism 2022; 26: 782–97. 34961364 10.1177/13623613211065542 PMC9008560

[14] Johnson NL , Salowitz N , Van Abel M , Dolan B , Van Hecke A , Ahamed SI. Autism and research using magnetic resonance imaging. J Radiol Nurs 2017; 36: 245–52.

[15] Nordahl CW , Mello M , Shen AM , Shen MD , Vismara LA , Li D , et al. Methods for acquiring MRI data in children with autism spectrum disorder and intellectual impairment without the use of sedation. J Neurodev Disord 2016; 8: 20. 27158271 10.1186/s11689-016-9154-9 PMC4858915

[16] Smith CJ , Bhanot A , Norman E , Mullett JE , Bilbo SD , McDougle CJ , et al. A protocol for sedation free MRI and PET imaging in adults with autism spectrum disorder. J Autism Dev Disord 2019; 49: 3036–44. 31004246 10.1007/s10803-019-04010-3

[17] Simhal AK , Filho JOA , Segura P , Cloud J , Petkova E , Gallagher R , et al. Predicting multiscan MRI outcomes in children with neurodevelopmental conditions following MRI simulator training. Dev Cogn Neurosci 2021; 52: 101009. 34649041 10.1016/j.dcn.2021.101009 PMC8517836

[18] Gilmore AD , Buser NJ , Hanson JL. Variations in structural MRI quality significantly impact commonly used measures of brain anatomy. Brain Inform 2021; 8: 7. 33860392 10.1186/s40708-021-00128-2 PMC8050166

[19] Chen M-H , Huang C-F , Lin Y-S , Chiu Y-N , Gau SS-F , Wu Y-Y. Validation of the Mandarin Chinese version of the Autism Diagnostic Observation Schedule-2 for autism spectrum disorder. Res Autism Spectr Disord 2023; 105: 102184.

[20] Huang CF , Lin YS , Chiu YN , Gau SS , Chen VC , Lin CF , et al. Validation of the Chinese version of the autism diagnostic interview-revised in autism spectrum disorder. Neuropsychiatr Dis Treat 2022; 18: 327–39. 35210779 10.2147/NDT.S345568 PMC8863335

[21] Hus V , Gotham K , Lord C. Standardizing ADOS domain scores: separating severity of social affect and restricted and repetitive behaviors. J Autism Dev Disord 2014; 44: 2400–12. 23143131 10.1007/s10803-012-1719-1 PMC3612387

[22] Gotham K , Pickles A , Lord C. Standardizing ADOS scores for a measure of severity in autism spectrum disorders. J Autism Dev Disord 2009; 39: 693–705. 19082876 10.1007/s10803-008-0674-3 PMC2922918

[23] Chen YL , Shen LJ , Gau SS. The Mandarin version of the Kiddie-Schedule for Affective Disorders and Schizophrenia-Epidemiological version for DSM-5 – a psychometric study. J Formos Med Assoc 2017; 116: 671–8. 28709821 10.1016/j.jfma.2017.06.013

[24] Wechsler D. Wechsler Intelligence Scale for Children – Fourth Edition (WISC-IV). Pearson, 2003.

[25] Wechsler D. Wechsler Adult Intelligence Scale – Fourth Edition (WAIS-IV). Pearson, 2008.

[26] Roid GH , Miller LJ. Leiter International Performance Scale-Revised (Leiter-R). Stoelting, 1997.

[27] Sparrow SS , Balla DA , Cicchetti DV. The Vineland Adaptive Behavior Scales: Interview Edition. American Guidance Service, 1984.

[28] Gioia GA , Isquith PK , Guy SC , Kenworthy L. Test review behavior rating inventory of executive function. Child Neuropsychol 2000; 6: 235–8. 11419452 10.1076/chin.6.3.235.3152

[29] Gau SS-F , Liu L-T , Wu Y-Y , Chiu Y-N , Tsai W-C. Psychometric properties of the Chinese version of the social responsiveness scale. Res Autism Spectr Disord 2013; 7: 349–60.

[30] Bodfish JW , Symons FJ , Parker DE , Lewis MH. Varieties of repetitive behavior in autism: comparisons to mental retardation. J Autism Dev Disord 2000; 30: 237–43. 11055459 10.1023/a:1005596502855

[31] McIntosh D , Miller L , Shyu V , Dunn W. Development and validation of the short sensory profile. Sensory Profile Manual 1999; 61: 59–73.

[32] Aman MG , Singh NN , Stewart AW , Field CJ. Psychometric characteristics of the aberrant behavior checklist. Am J Ment Defic 1985; 89: 492–502. 3158201

[33] Rong Y , Yang C-J , Jin Y , Wang Y. Prevalence of attention-deficit/hyperactivity disorder in individuals with autism spectrum disorder: a meta-analysis. Res Autism Spectr Disord 2021; 83: 101759.

[34] Gau SS , Shang CY , Liu SK , Lin CH , Swanson JM , Liu YC , et al. Psychometric properties of the Chinese version of the Swanson, Nolan, and Pelham, version IV scale – parent form. Int J Methods Psychiatr Res 2008; 17: 35–44. 18286459 10.1002/mpr.237 PMC6878250

[35] Barnevik Olsson M , Holm A , Westerlund J , Lundholm Hedvall Å , Gillberg C , Fernell E. Children with borderline intellectual functioning and autism spectrum disorder: developmental trajectories from 4 to 11 years of age. Neuropsychiatr Dis Treat 2017; 13: 2519–26. 29042781 10.2147/NDT.S143234 PMC5634384

[36] Alvares GA , Bebbington K , Cleary D , Evans K , Glasson EJ , Maybery MT , et al. The misnomer of ‘high functioning autism’: intelligence is an imprecise predictor of functional abilities at diagnosis. Autism 2020; 24: 221–32. 31215791 10.1177/1362361319852831

[37] Xu J , Moeller S , Auerbach EJ , Strupp J , Smith SM , Feinberg DA , et al. Evaluation of slice accelerations using multiband echo planar imaging at 3 T. NeuroImage 2013; 83: 991–1001. 23899722 10.1016/j.neuroimage.2013.07.055 PMC3815955

[38] Gabrielsen TP , Anderson JS , Stephenson KG , Beck J , King JB , Kellems R , et al. Functional MRI connectivity of children with autism and low verbal and cognitive performance. Mol Autism 2018; 9: 67. 30603063 10.1186/s13229-018-0248-y PMC6307191

[39] Vanderwal T , Kelly C , Eilbott J , Mayes LC , Castellanos FX. Inscapes: a movie paradigm to improve compliance in functional magnetic resonance imaging. NeuroImage 2015; 122: 222–32. 26241683 10.1016/j.neuroimage.2015.07.069 PMC4618190

[40] Gaser C , Dahnke R , Thompson PM , Kurth F , Luders E. The Alzheimer’s Disease Neuroimaging I. CAT: a computational anatomy toolbox for the analysis of structural MRI data. GigaScience 2024; 13: giae049. 39102518 10.1093/gigascience/giae049 PMC11299546

[41] Marcus DS , Harms MP , Snyder AZ , Jenkinson M , Wilson JA , Glasser MF , et al. Human Connectome Project informatics: quality control, database services, and data visualization. NeuroImage 2013; 80: 202–19. 23707591 10.1016/j.neuroimage.2013.05.077 PMC3845379

[42] DuPre E , Salo T , Ahmed Z , Bandettini PA , Bottenhorn KL , Caballero-Gaudes C , et al. TE-dependent analysis of multi-echo fMRI with* tedana. J Open Source Softw 2021; 6: 3669.

[43] Kundu P , Brenowitz ND , Voon V , Worbe Y , Vértes PE , Inati SJ , et al. Integrated strategy for improving functional connectivity mapping using multiecho fMRI. Proc Natl Acad Sci USA 2013; 110: 16187–92. 24038744 10.1073/pnas.1301725110 PMC3791700

[44] Kundu P , Inati SJ , Evans JW , Luh WM , Bandettini PA. Differentiating BOLD and non-BOLD signals in fMRI time series using multi-echo EPI. NeuroImage 2012; 60: 1759–70. 22209809 10.1016/j.neuroimage.2011.12.028 PMC3350785

[45] Kundu P , Voon V , Balchandani P , Lombardo MV , Poser BA , Bandettini PA. Multi-echo fMRI: a review of applications in fMRI denoising and analysis of BOLD signals. NeuroImage 2017; 154: 59–80. 28363836 10.1016/j.neuroimage.2017.03.033

[46] Lin HY , Tseng WY , Lai MC , Matsuo K , Gau SS. Altered resting-state frontoparietal control network in children with attention-deficit/hyperactivity disorder. J Int Neuropsychol Soc 2015; 21: 271–84. 25928822 10.1017/S135561771500020X

[47] Power JD , Barnes KA , Snyder AZ , Schlaggar BL , Petersen SE. Spurious but systematic correlations in functional connectivity MRI networks arise from subject motion. NeuroImage 2012; 59: 2142–54. 22019881 10.1016/j.neuroimage.2011.10.018 PMC3254728

[48] Yan C-G , Cheung B , Kelly C , Colcombe S , Craddock RC , Di Martino A , et al. A comprehensive assessment of regional variation in the impact of head micromovements on functional connectomics. NeuroImage 2013; 76: 183–201. 23499792 10.1016/j.neuroimage.2013.03.004 PMC3896129

[49] Pua EPK , Barton S , Williams K , Craig JM , Seal ML. Individualised MRI training for paediatric neuroimaging: a child-focused approach. Dev Cogn Neurosci 2020; 41: 100750. 31999567 10.1016/j.dcn.2019.100750 PMC6994628

[50] Yeh CH , Tseng RY , Ni HC , Cocchi L , Chang JC , Hsu MY , et al. White matter microstructural and morphometric alterations in autism: implications for intellectual capabilities. Mol Autism 2022; 13: 21. 35585645 10.1186/s13229-022-00499-1 PMC9118608

[51] Tournier JD , Smith R , Raffelt D , Tabbara R , Dhollander T , Pietsch M , et al. MRtrix3: a fast, flexible and open software framework for medical image processing and visualisation. NeuroImage 2019; 202: 116137. 31473352 10.1016/j.neuroimage.2019.116137

[52] Smith SM , Jenkinson M , Woolrich MW , Beckmann CF , Behrens TE , Johansen-Berg H , et al. Advances in functional and structural MR image analysis and implementation as FSL. NeuroImage 2004; 23: S208–19. 15501092 10.1016/j.neuroimage.2004.07.051

[53] Glickman ME , Rao SR , Schultz MR. False discovery rate control is a recommended alternative to Bonferroni-type adjustments in health studies. J Clin Epidemiol 2014; 67: 850–7. 24831050 10.1016/j.jclinepi.2014.03.012

[54] Greene DJ , Koller JM , Hampton JM , Wesevich V , Van AN , Nguyen AL , et al. Behavioral interventions for reducing head motion during MRI scans in children. NeuroImage 2018; 171: 234–45. 29337280 10.1016/j.neuroimage.2018.01.023 PMC5857466

[55] Chaudhary K , Bagharwal P , Wadhawan S. Anesthesia for intellectually disabled. J Anaesthesiol Clin Pharmacol 2017; 33: 432–40. 29416231 10.4103/joacp.JOACP_357_15 PMC5791252

[56] Chen X , Zheng X , Cai J , Yang X , Lin Y , Wu M , et al. Effect of anesthetics on functional connectivity of developing brain. Front Hum Neurosci 2022; 16: 853816. 35360283 10.3389/fnhum.2022.853816 PMC8963106

[57] Feinberg DA , Yacoub E. The rapid development of high speed, resolution and precision in fMRI. NeuroImage 2012; 62: 720–5. 22281677 10.1016/j.neuroimage.2012.01.049 PMC3389295

[58] Phạm DĐ. , McDonald DJ , Ding L , Nebel MB , Mejia AF. Less is more: balancing noise reduction and data retention in fMRI with data-driven scrubbing. NeuroImage 2023; 270: 119972. 36842522 10.1016/j.neuroimage.2023.119972 PMC10773988

[59] Beckers AB , Drenthen GS , Jansen JFA , Backes WH , Poser BA , Keszthelyi D. Comparing the efficacy of data-driven denoising methods for a multi-echo fMRI acquisition at 7T. NeuroImage 2023; 280: 120361. 37669723 10.1016/j.neuroimage.2023.120361

[60] Di Martino A , O.’Connor D , Chen B , Alaerts K , Anderson JS , Assaf M , et al. Enhancing studies of the connectome in autism using the autism brain imaging data exchange II. Sci Data 2017; 4: 170010. 28291247 10.1038/sdata.2017.10 PMC5349246

[61] Harkness K , Bray S , Murias K. The role of stimulant washout status in functional connectivity of default mode and fronto-parietal networks in children with neurodevelopmental conditions. Res Dev Disabil 2024; 146: 104691. 38340416 10.1016/j.ridd.2024.104691

[62] Lai MC , Lin HY , Ameis SH. Towards equitable diagnoses for autism and attention-deficit/hyperactivity disorder across sexes and genders. Curr Opin Psychiatry 2022; 35: 90–100. 35084380 10.1097/YCO.0000000000000770

