# Supplementary material for: Factors linked to successful brain magnetic resonance imaging scans and data quality in autistic individuals across the functioning spectrum
Source: BJPsych Open. 2026 Jul 14;12(4):e186. doi: 10.1192/bjo.2026.12035 (PMC13372812; doi:10.1192/bjo.2026.12035)
Supplement: Huang et al. supplementary material [file S2056472426120353sup001.docx]

**Supplementary Information**

**Factors linked to successful brain MRI scans and data quality in autistic individuals**

Lin-Wan Huang, Yi Ran Zhou, Chun-Hung Yeh, Hsing-Chang Ni, Benoit H Mulsant, Muhammad Ishrat Husain, Jung-Chi Chang, En-Nien Tu, Mei-Yun Hsu, Yu-Yu Wu, Tai-Li Chou, Susan Shur-Fen Gau, Hsiang-Yuan Lin

**STable 1.** *Co-occurring neuropsychiatric conditions and Medication Use for ASC-IA & ASC-II.*

|  | ASC-IA (n= 37) | ASC-II (n= 46) |
| --- | --- | --- |
|  | | |
| No co-occurring neuropsychiatric conditions | 10 | 12 |
| Co-occurring ADHD | 21 | 25 |
| Co-occurring Anxiety disorder | 8 (5 social anxiety disorder; 1 history of mild agoraphobia; 1 height phobia) | 12 (7 specific phobia; 5 social anxiety disorder; 1 selective mutism) |
| Co-occurring tic disorder | 3 | 6 (2 Tourette syndrome) |
| Co-occurring OCD | 1 | 1 |
| Co-occurring learning disorder | 1 (writing/reading disorder) | N/A |
| Co-occurring depressive disorder | 3 (1 history of depression; 1 dysthymic disorder & 1 mild MDD) | 2 (history of depression) |
| Other co-occurring conditions | 1 gender dysphoria  1 ODD | 3 epilepsy (well controlled)  2 ODD |
|  |  |  |
| **Medications** | | |
| Methylphenidate | 13 | 9 |
| Antidepressant | 2 (1 sertraline; 1 fluoxetine) | 2 (sertraline; 1 fluoxetine) |
| Valproic acid (mood stabilizer) | N/A | 3 |
| Antipsychotic | N/A | 2 |

*Abbreviations:* ADHD = Attention-Deficit/Hyperactivity Disorder; MDD = Major Depressive Disorder; OCD = Obsessive-Compulsive Disorder; ODD = Oppositional Defiant Disorder.

| **STable 2.** *Demographic and clinical profiles of participants (Grouping 1).* | | |  |  |
| --- | --- | --- | --- | --- |
|  | ASC (*n*=83) | TDC (*n*=39) | Statistics^a^ |  |
| **Demographics** |  |  |  |  |
| Age | 15 [9] | 17 [8] | *H* = 0.75, *p =* 0.538 |  |
| Range | 7-30 | 8-30 |  |  |
| Sex (male/female) | 74/9 | 31/8 | *X^2^* = 2.07, *p =* 0.150 |  |
| Medication (yes/no) | 44/39 | 0/39 | *X^2^* = 32.34*** |  |
| Comorbidity (yes/no) | 55/28 | 0/39 | *X^2^* = 47.06*** |  |
| **ASC Profile** |  |  |  |  |
| ADOS-2 CSS^b^ | 6 [3] | — | — |  |
| ADI-R Total | 23 [8] | — | — |  |
| SRS Total | 94 [33.5] | 19 [12] | *H* = 72.28*** |  |
| SSP Total | 31 [31] | 3 [8] | *H* = 58.78*** |  |
| RBS-R Total | 24.5 [24.8] | 1 [5] | *H* = 61.20*** |  |
| **Functional Profile** |  |  |  |  |
| Wechsler FIQ^c^ | 84 [35.5] | 113 [15] | *H* = 38.41*** |  |
| Leiter-R NVFIQ | 97.5 [49.2] | 121 [12] | *H* = 29.57*** |  |
| VABS ABC | 73 [28.8] | 111.5 [25.8] | *H* = 61.24*** |  |
| BRIEF-GEC | 155 [38.5] | 87 [27] | *H* = 65.97*** |  |
| **Behavioral Profile** |  |  |  |  |
| ABC Total | 46 [53] | 0 [5] | *H* = 60.11*** |  |
| SNAP-IV Total | 22 [17] | 3 [9] | *H* = 55.46*** |  |
| **MRI Quality Metrics** |  |  |  |  |
| sMRI – IQR | 88.32 [1.53] | 88.98 [0.50] | *H* = 23.81*** |  |
| fMRI – Mean FD (mm) | 0.50 [0.59] | 0.26 [0.30] | *H* = 9.65, *p =* 0.002 |  |
| fMRI – A/R component ratio | 0.14 [0.18] | 0.22 [0.18] | *H* = 1.57, *p =* 0.211 |  |
| dMRI – Mean Relative RMS (mm) | 0.37 [0.32] | 0.32 [0.17] | *H* = 1.21, *p =* 0.271 |  |
| *Note.* All continuous variables are presented as Median [Interquartile Range]. ***p < 0.001. *Abbreviations:* ASC = intellectually-able autism spectrum condition and autism spectrum condition with intellectual impairment only and autism spectrum condition with intellectual impairment and minimally verbal status; TDC = typically developing control; ABC Total = Aberrant Behavior Checklist, Total Score; ADI-R Total = Autism Diagnostic Interview-Revised, Total Score; ADOS-2 CSS = Autism Diagnostic Observation Schedule-Second Edition, Calibrated Severity Score; BRIEF-GEC = Behavior Rating Inventory of Executive Function, Global Executive Composite; Leiter-R NVFIQ = Leiter International Performance Scale-Revised, Non-verbal Full Intelligence Quotient; RBS-R Total = Repetitive Behavior Scale-Revised, Total Score; SNAP-IV Total = Swanson, Nolan, and Pelham-IV Questionnaire, Total Score; SRS Total = Social Responsiveness Scale, Total Score; SSP Total = Short Sensory Profile, Total Score; VABS ABC = Vineland Adaptive Behavior Scale, Adaptive Behavior Composite; Wechsler FIQ = Wechsler Intelligence Scale, Full-scale Intelligence Quotient. ^a^ Chi-square test was used for sex, medication, and comorbidity variables; Kruskal-Wallis test was used for all other variables. ^b^ An appropriate ADOS-2 module was selected for each participant as determined by a child psychiatrist. ^c^ Wechsler Adult Intelligence Scale, Fourth Edition was used for participants ages 16 and above while Wechsler Intelligence Scale for Children, Fourth Edition was used for participants less than 16 years old. | | | |  |
|  |  |  |  |  |
|  |  |  |  |  |
|  |  |  |  |  |
|  |  |  |  |  |
|  |  |  |  |  |
|  |  |  |  |  |
|  |  |  |  |  |
|  |  |  |  |  |
|  |  |  |  |  |
|  |  |  |  |  |
|  |  |  |  |  |
|  |  |  |  |  |
|  |  |  |  |  |
|  |  |  |  |  |
|  |  |  |  |  |
|  |  |  |  |  |
|  |  |  |  |  |
|  |  |  |  |  |

| **STable 3.** *Demographic and clinical profiles of participants (Grouping 3).* | | | |  |  |  |  |
| --- | --- | --- | --- | --- | --- | --- | --- |
|  | ASC-MV (*n=19*) | ASC-IIO (*n*=27) | ASC-IA (*n*=37) | TDC (*n*=39) | Statistics^a^ | Post-hoc^b^ |  |
| **Demographics** |  |  |  |  |  |  |  |
| Age | 15 [12] | 16 [9] | 15 [9] | 17 [8] | *H* = 1.24, *p =* 0.743 | — |  |
| Range | 7-28 | 7-30 | 7-26 | 8-30 |  |  |  |
| Sex (male/female) | 19/0 | 23/4 | 32/5 | 31/8 | n.s.^e^ | — |  |
| Medication (yes/no) | 9/10 | 14/13 | 21/16 | 0/39 | *X^2^* = 32.84*** | ASC-MV, ASC-IIO, ASC-IA > TDC |  |
| Comorbidity (yes/no) | 12/7 | 19/8 | 24/13 | 0/39 | *X^2^* = 47.34*** | ASC-MV, ASC-IIO, ASC-IA > TDC |  |
| **ASC Profile** |  |  |  |  |  |  |  |
| ADOS-2 CSS^c^ | 5 [2] | 7 [3] | 5 [4] | — | *H = 15.76**** | ASC-IIO > ASC-MV, ASC-IA |  |
| ADI-R Total | 27 [6] | 25 [6] | 21 [8] | — | *H* = 12.96** | ASC-MV > ASC-IA |  |
| SRS Total | 110.5 [31] | 93.5 [32.8] | 85 [35.5] | 19 [12] | *H* = 77.58*** | ASC-MV, ASC-IIO, ASC-IA > TDC |  |
| SSP Total | 39.5 [23] | 29.5 [34.8] | 30 [35.5] | 3 [8] | *H* = 60.02*** | ASC-MV, ASC-IIO, ASC-IA > TDC |  |
| RBS-R Total | 30.5 [28] | 17 [24.8] | 24.5 [24.5] | 1 [5] | *H* = 62.46*** | ASC-MV, ASC-IIO, ASC-IA > TDC |  |
| **Functional Profile** |  |  |  |  |  |  |  |
| Wechsler FIQ^d^ | 46 [21] | 71 [11.5] | 102 [17.8] | 113 [15] | *H* = 78.20*** | ASC-MV, ASC-IIO < ASC-IA, TDC |  |
| Leiter-R NVFIQ | 58 [38.5] | 87 [35] | 119 [23] | 121 [12] | *H* = 67.37*** | ASC-MV, ASC-IIO < ASC-IA, TDC |  |
| VABS ABC | 56.5 [7] | 68 [11] | 90 [21] | 111.5 [25.8] | *H* = 90.06*** | ASC-MV, ASC-IIO < ASC-IA < TDC |  |
| BRIEF-GEC | 168.5 [43.5] | 153 [41.8] | 155 [30] | 87 [27] | *H* = 66.22*** | ASC-MV, ASC-IIO, ASC-IA > TDC |  |
| **Behavioral Profile** |  |  |  |  |  |  |  |
| ABC Total | 63.5 [49.5] | 35.1 [58.8] | 34 [43] | 0 [5] | *H* = 61.67*** | ASC-MV, ASC-IIO, ASC-IA > TDC |  |
| SNAP-IV Total | 27 [13.5] | 19 [17.5] | 22 [17.5] | 3 [9] | *H* = 56.87*** | ASC-MV, ASC-IIO, ASC-IA > TDC |  |
| **MRI Quality Metrics** |  |  |  |  |  |  |  |
| sMRI – IQR | 88.03 [1.40] | 88.38 [0.96] | 88.34 [1.59] | 88.98 [0.50] | *H* = 24.64*** | ASC-MV, ASC-IIO, ASC-IA < TDC |  |
| fMRI – Mean FD (mm) | 0.50 [1.35] | 0.45 [0.64] | 0.50 [0.55] | 0.26 [0.30] | *H* = 12.03, *p =* 0.007 | ASC-MV > TDC |  |
| fMRI – A/R component ratio | 0.12 [0.23] | 0.12 [0.19] | 0.15 [0.18] | 0.22 [0.18] | *H* = 1.77, *p =* 0.622 | — |  |
| dMRI – Mean Relative RMS (mm) | 0.34 [0.16] | 0.47 [0.39] | 0.35 [0.22] | 0.32 [0.17] | *H* = 3.72, *p =* 0.293 | — |  |
| *Note.* All continuous variables are presented as Median [Interquartile Range]. ***p* < 0.01, ***p < 0.001, n.s. = not significant (p > 0.05). *Abbreviations:* ASC-MV = autism spectrum condition with intellectual impairment and minimally verbal status, ASC-IIO = autism spectrum condition with intellectual impairment only; ASC-IA = intellectually-able autism spectrum condition; TDC = typically developing control; ABC Total = Aberrant Behavior Checklist, Total Score; ADI-R Total = Autism Diagnostic Interview-Revised, Total Score; ADOS-2 CSS = Autism Diagnostic Observation Schedule-Second Edition, Calibrated Severity Score; BRIEF-GEC = Behavior Rating Inventory of Executive Function, Global Executive Composite; Leiter-R NVFIQ = Leiter International Performance Scale-Revised, Non-verbal Full Intelligence Quotient; RBS-R Total = Repetitive Behavior Scale-Revised, Total Score; SNAP-IV Total = Swanson, Nolan, and Pelham-IV Questionnaire, Total Score; SRS Total = Social Responsiveness Scale, Total Score; SSP Total = Short Sensory Profile, Total Score; VABS ABC = Vineland Adaptive Behavior Scale, Adaptive Behavior Composite; Wechsler FIQ = Wechsler Intelligence Scale, Full-scale Intelligence Quotient. ^a^ Chi-square test was used for sex, medication, and comorbidity variables; Kruskal-Wallis test was used for all other variables. ^b^ Pairwise comparisons using chi-square test followed by Bonferonni correction was performed for sex, medication, and comorbidity variables; post-hoc Dunn-Bonferroni test was used for all other variables. ^c^ An appropriate ADOS-2 module was selected for each participant as determined by a child psychiatrist. ^d^ Wechsler Adult Intelligence Scale, Fourth Edition was used for participants ages 16 and above while Wechsler Intelligence Scale for Children, Fourth Edition was used for participants less than 16 years old. ^e^ Did not meet criteria for chi-square test, Fisher's exact test suggested no significant differences between groups. | | | | | | |  |
|  |  |  |  |  |  |  |  |
|  |  |  |  |  |  |  |  |
|  |  |  |  |  |  |  |  |
|  |  |  |  |  |  |  |  |
|  |  |  |  |  |  |  |  |
|  |  |  |  |  |  |  |  |
|  |  |  |  |  |  |  |  |
|  |  |  |  |  |  |  |  |
|  |  |  |  |  |  |  |  |
|  |  |  |  |  |  |  |  |
|  |  |  |  |  |  |  |  |
|  |  |  |  |  |  |  |  |
|  |  |  |  |  |  |  |  |
|  |  |  |  |  |  |  |  |

| **STable 4.** *Comparison of demographic and clinical variables between successful and unsuccessful scans in autism spectrum condition (ASC) and typically developing controls (TDC) participants (Grouping 1).* | | | | |  |
| --- | --- | --- | --- | --- | --- |
|  | **ASC (*n*=83)** | | **TDC (*n*=39)** | |  |
|  | **Successful (n=46)** | **Statistics** | **Successful (n=33)** | **Statistics** |  |
|  | **Not Successful (n=37)** |  | **Not Successful (n=6)** |  |  |
| **Demographics** | | | | |  |
| Age | 18 [9] | *U* = 457.5, *q* = 0.010 | 18 [7] | *U* = 13.5, *q <* 0.001 |  |
|  | 11 [7] |  | 9.5 [3] |  |  |
| **ASC Profile** | | | | |  |
| ADOS-2 CSS | 5 [3] | *U* = 751, *q* = 0.412 | — | |  |
|  | 6 [4] |  |  |  |  |
| ADI-R Total | 22 [10] | *U* = 688, *q* = 0.043 | — | |  |
|  | 25 [8] |  |  |  |  |
| SRS Total | 93 [34] | *U* = 684.5, *q* = 0.877 | 18 [13] | *U* = 73, *q* = 0.346 |  |
|  | 94 [39] |  | 21 [17] |  |  |
| SSP Total | 29 [38] | *U* = 631.5, *q* = 0.294 | 3 [8] | *U* = 98.5, *q* = 0.899 |  |
|  | 39 [22] |  | 2 [17] |  |  |
| RBS-R Total | 16 [36] | *U* = 591.5, *q* = 0.392 | 1 [4] | *U* = 56.5, *q* = 0.448 |  |
|  | 28 [14] |  | 4 [9] |  |  |
| **Functional Profile** | | | | |  |
| Wechsler FIQ^b^ | 86 [33] | *U* = 507, *q* = 0.112 | 113 [15] | *U* = 88, *q* = 0.782 |  |
|  | 73 [38] |  | 110 [22] |  |  |
| Leiter-R NVFIQ | 101 [46] | *U* = 651, *q* = 0.017 | 123 [12] | *U* = 50.5, *q* = 0.021 |  |
|  | 93 [44] |  | 110 [21] |  |  |
| VABS ABC | 75 [28] | *U* = 661.5, *q* = 0.032 | 113 [32] | *U* = 61, *q* = 0.448 |  |
|  | 71 [25] |  | 106 [31] |  |  |
| BRIEF-GEC | 154 [44] | *U* = 632, *q* = 0.410 | 84 [17] | *U* = 18, *q <* 0.001 |  |
|  | 164 [33] |  | 117 [27] |  |  |
| **Behavioral Profile** | | | | |  |
| ABC Total | 37 [42] | *U* = 599.5, *q* = 0.503 | 0 [3] | *U* = 42.5, *q* = 0.340 |  |
|  | 58 [57] |  | 8 [13] |  |  |
| SNAP-IV Total | 21 [15] | *U* = 499, *q* = 0.048 | 2 [6] | *U* = 16, *q <* 0.001 |  |
|  | 28 [18] |  | 12 [5] |  |  |
| *Note.* Scans successful if participant completed T1, rsfMRI, and dMRI. All continuous variables are presented as Median (IQR). | | | | |  |
| *Abbreviations:* ASC = intellectually-able autism spectrum condition and autism spectrum condition with intellectual impairment only and autism spectrum condition with intellectual impairment and minimally verbal status; TDC = typically developing control; ABC Total = Aberrant Behavior Checklist, Total Score; ADI-R Total = Autism Diagnostic Interview-Revised, Total Score; ADOS-2 CSS = Autism Diagnostic Observation Schedule-Second Edition, Calibrated Severity Score; BRIEF-GEC = Behavior Rating Inventory of Executive Function, Global Executive Composite; Leiter-R NVFIQ = Leiter International Performance Scale-Revised, Non-verbal Full Intelligence Quotient; RBS-R Total = Repetitive Beheavior Scale-Revised, Total Score; SNAP-IV Total = Swanson, Nolan, and Pelham-IV Questionnaire, Total Score; SRS Total = Social Responsiveness Scale, Total Score; SSP Total = Short Sensory Profile, Total Score; VABS ABC = Vineland Adaptive Behavior Scale, Adaptive Behavior Composite; Wechsler FIQ = Wechsler Intelligence Scale, Full-scale Intelligence Quotient. ^a^ Mann-Whitney U test was used followed by the Benjamini-Hochberg procedure to give False Discovery Rate (FDR) adjusted p-values (q-values), calculated separately for each diagnostic categories. ^b^ Wechsler Adult Intelligence Scale, Fourth Edition was used for participants ages 16 and above while Wechsler Intelligence Scale for Children, Fourth Edition was used for participants less than 16 years old. | | | | |  |
|  |  |  |  |  |  |
|  |  |  |  |  |  |
|  |  |  |  |  |  |
|  |  |  |  |  |  |
|  |  |  |  |  |  |
|  |  |  |  |  |  |
|  |  |  |  |  |  |
|  |  |  |  |  |  |
|  |  |  |  |  |  |
|  |  |  |  |  |  |
|  |  |  |  |  |  |
|  |  |  |  |  |  |
|  |  |  |  |  |  |
|  |  |  |  |  |  |
|  |  |  |  |  |  |
|  |  |  |  |  |  |
|  |  |  |  |  |  |
|  |  |  |  |  |  |
|  |  |  |  |  |  |
|  |  |  |  |  |  |
|  |  |  |  |  |  |

| **STable 5.** *Comparison of demographic and clinical variables between successful and unsuccessful scans in autism spectrum condition (ASC) and typically developing controls (TDC) participants (Grouping 3).* | | | | | | | | |  |
| --- | --- | --- | --- | --- | --- | --- | --- | --- | --- |
|  | **ASC-MV (*n*=19)** | | **ASC-IIO (*n*=27)** | | **ASC-IA (*n*=37)** | | **TDC (*n*=39)** | |  |
|  | **Successful (n=5)** | **Statistics** | **Successful (n=17)** | **Statistics** | **Successful (n=24)** | **Statistics** | **Successful (n=33)** | **Statistics** |  |
|  | **Not Successful (n=14)** |  | **Not Successful (n=10)** |  | **Not Successful (n=13)** |  | **Not Successful (n=6)** |  |  |
| **Demographics** | | | | | | | | |  |
| Age | 23 [5] | *U* = 9, *q* = 0.298 | 16 [9] | *U* = 66.5, *q* = 0.881 | 17.5 [8] | *U* = 59, *q* = 0.022 | 18 [7] | *U* = 13.5, *q <* 0.001 |  |
|  | 12 [8] |  | 14 [7] |  | 10 [3] |  | 9.5 [3] |  |  |
| **ASC Profile** | | | | | | | | |  |
| ADOS-2 CSS | 5 [1] | *U* = 27.5, *q* = 0.704 | 7 [4] | *U* = 62.5, *q* = 0.881 | 5 [3] | *U* = 130, *q* = 0.590 | — | |  |
|  | 6 [2] |  | 8 [3] |  | 5 [3] |  |  |  |  |
| ADI-R Total | 25 [6] | *U* = 28.5, *q* = 0.704 | 27 [8] | *U* = 60.5, *q* = 0.881 | 20 [8] | *U* = 111, *q* = 0.590 | — | |  |
|  | 27 [9] |  | 23 [5] |  | 22 [9] |  |  |  |  |
| SRS Total | 112 [52] | *U* = 30.5, *q* = 0.704 | 93 [27] | *U* = 75, *q* = 1 | 86 [38] | *U* = 127, *q* = 0.929 | 18 [13] | *U* = 73, *q* = 0.346 |  |
|  | 109 [32] |  | 94 [43] |  | 84 [38] |  | 21 [17] |  |  |
| SSP Total | 55 [77] | *U = 27*, *q* = 0.701 | 30 [31] | *U* = 69, *q* = 1 | 26 [22] | *U* = 91.5, *q* = 0.280 | 3 [8] | *U* = 98.5, *q* = 0.899 |  |
|  | 39 [19] |  | 29 [39] |  | 40 [31] |  | 2 [17] |  |  |
| RBS-R Total | 45 [50] | *U = 30*, *q* = 0.701 | 16 [30] | *U* = 76, *q* = 1 | 15 [29] | *U* = 87.5, *q* = 0.280 | 1 [4] | *U* = 56.5, *q* = 0.448 |  |
|  | 30 [15] |  | 20 [21] |  | 28 [20] |  | 4 [9] |  |  |
| **Functional Profile** | | | | | | | | |  |
| Wechsler FIQ^b^ | 41 [.]^C^ | *U* = 8, *q* = 0.701 | 73 [15] | *U* = 58, *q* = 0.881 | 104 [18] | *U* = 149, *q* = 0.928 | 113 [15] | *U* = 88, *q* = 0.782 |  |
|  | 51 [26] |  | 71 [6] |  | 99 [27] |  | 110 [22] |  |  |
| Leiter-R NVFIQ | 47 [49] | *U* = 25, *q* = 0.704 | 87 [40] | *U* = 83.5, *q* = 1 | 121 [25] | *U* = 121, *q* = 0.280 | 123 [12] | *U* = 50.5, *q* = 0.021 |  |
|  | 63 [41] |  | 86 [25] |  | 111 [20] |  | 110 [21] |  |  |
| VABS ABC | 56 [12] | *U* = 28.5, *q* = 0.701 | 68 [10] | *U* = 46, *q* = 0.625 | 91 [20] | *U* = 131.5, *q* = 0.907 | 113 [32] | *U* = 61, *q* = 0.448 |  |
|  | 57 [7] |  | 73.5 [10] |  | 89 [24] |  | 106 [31] |  |  |
| BRIEF-GEC | 178 [61] | *U* = 28.5, *q* = 0.701 | 153 [38] | *U* = 72.5, *q* = 1 | 154 [39] | *U* = 89.5, *q* = 0.310 | 84 [17] | *U* = 18, *q <* 0.001 |  |
|  | 167 [45] |  | 162 [44] |  | 164 [34] |  | 117 [27] |  |  |
| **Behavioral Profile** | | | | | | | | |  |
| ABC Total | 46 [63] | *U* = 20, *q* = 0.704 | 38 [50] | *U* = 104, *q* = 1 | 35 [41] | *U* = 113.5, *q* = 0.590 | 0 [3] | *U* = 42.5, *q* = 0.340 |  |
|  | 70 [39] |  | 27 [79] |  | 32 [49] |  | 8 [13] |  |  |
| SNAP-IV Total | 23 [29] | *U* = 22.5, *q* = 0.862 | 21 [14] | *U* = 74.5, *q* = 1 | 21 [12] | *U* = 80.5, *q* = 0.280 | 2 [6] | *U* = 16, *q <* 0.001 |  |
|  | 27 [11] |  | 17 [25] |  | 28 [22] |  | 12 [5] |  |  |
| *Note.* Scans successful if participant completed T1, rsfMRI, and dMRI. All continuous variables are presented as Median [Interquartile Range]. | | | | | | | | |  |
| *Abbreviations:* ASC-II = autism spectrum condition with intellectual impairment only or autism spectrum condition with intellectual impairment and minimally verbal status; ASC-IA = intellectually-able autism spectrum condition; TDC = typically developing control; ABC Total = Aberrant Behavior Checklist, Total Score; ADI-R Total = Autism Diagnostic Interview-Revised, Total Score; ADOS-2 CSS = Autism Diagnostic Observation Schedule-Second Edition, Calibrated Severity Score; BRIEF-GEC = Behavior Rating Inventory of Executive Function, Global Executive Composite; Leiter-R NVFIQ = Leiter International Performance Scale-Revised, Non-verbal Full Intelligence Quotient; RBS-R Total = Repetitive Beheavior Scale-Revised, Total Score; SNAP-IV Total = Swanson, Nolan, and Pelham-IV Questionnaire, Total Score; SRS Total = Social Responsiveness Scale, Total Score; SSP Total = Short Sensory Profile, Total Score; VABS ABC = Vineland Adaptive Behavior Scale, Adaptive Behavior Composite; Wechsler FIQ = Wechsler Intelligence Scale, Full-scale Intelligence Quotient. ^a^ Mann-Whitney U test was used followed by the Benjamini-Hochberg procedure to give False Discovery Rate (FDR) adjusted p-values (q-values), calculated separately for each diagnostic categories. ^b^ Wechsler Adult Intelligence Scale, Fourth Edition was used for participants ages 16 and above while Wechsler Intelligence Scale for Children, Fourth Edition was used for participants less than 16 years old.  ^C^ Only three ASC-MV participants had completed Wechsler’s IQ assessments. | | | | | | | | |  |
|  |  |  |  |  |  |  |  |  |  |
|  |  |  |  |  |  |  |  |  |  |
|  |  |  |  |  |  |  |  |  |  |
|  |  |  |  |  |  |  |  |  |  |
|  |  |  |  |  |  |  |  |  |  |
|  |  |  |  |  |  |  |  |  |  |
|  |  |  |  |  |  |  |  |  |  |
|  |  |  |  |  |  |  |  |  |  |
|  |  |  |  |  |  |  |  |  |  |
|  |  |  |  |  |  |  |  |  |  |
|  |  |  |  |  |  |  |  |  |  |
|  |  |  |  |  |  |  |  |  |  |
|  |  |  |  |  |  |  |  |  |  |
|  |  |  |  |  |  |  |  |  |  |
|  |  |  |  |  |  |  |  |  |  |
|  |  |  |  |  |  |  |  |  |  |
|  |  |  |  |  |  |  |  |  |  |
|  |  |  |  |  |  |  |  |  |  |
|  |  |  |  |  |  |  |  |  |  |
|  |  |  |  |  |  |  |  |  |  |
